# Overexpression of PGC-1α influences the mitochondrial unfolded protein response (mtUPR) induced by MPP^+^ in human SH-SY5Y neuroblastoma cells

**DOI:** 10.1038/s41598-020-67229-6

**Published:** 2020-06-26

**Authors:** Yousheng Cai, Hui Shen, Huidan Weng, Yingqing Wang, Guoen Cai, Xiaochun Chen, Qinyong Ye

**Affiliations:** 1 Department of Neurology, Fujian Institute of Geriatrics, Fujian Medical University Union Hospital, 29 Xinquan Road, Fuzhou, Fujian 350001 China; 2 Department of Neurology, Zhangzhou Affiliated Hospital of Fujian Medical University, 59 Shengli Road, Zhangzhou, 363000 China; 3 Institute of Neuroscience, Fujian Key Laboratory of Molecular Neurology, Fujian Medical University, 29 Xinquan Road, Fuzhou, 350001 China

**Keywords:** Molecular neuroscience, Molecular neuroscience, Parkinson's disease, Parkinson's disease

## Abstract

Parkinson’s disease (PD) is a common dyskinesia disease, the mitochondrial unfolded protein response (mtUPR) may be directly or indirectly involved in the occurrence and development of PD, although the exact mechanism is unclear. We established a dopaminergic neuronal-like cell model of PD, by overexpression of PGC-1α to detect evaluate the expression of proteases and molecular chaperones of involved in the mtUPR, as well as the expression of PGC-1α and LRPPRC, illustrated the distribution of LRPPRC. Remarkably, the mtUPR activation reached maximal at 24 h after MPP^+^ treatment in SH-SY5Y cells, which the protein and transcription levels of the proteases and molecular chaperones reached maximal. The proteases and molecular chaperones were significantly increased when overexpressed PGC-1α, which indicated that PGC-1α overexpression activated the mtUPR, and PGC-1α had a protective effect on SH-SY5Y cells. The expression levels of PGC-1α and LRPPRC were significantly improved in the PGC-1α overexpression groups. LRPPRC was markedly reduced in the nucleus, suggesting that PGC-1α overexpression may play a protective role to the mitochondria through LRPPRC. Our finding indicates that overexpression of PGC-1α may activate mtUPR, reducing the oxidative stress injury induced by MPP^+^ through LRPPRC signaling, thus maintain mitochondrial homeostasis.

## Introduction

Parkinson’s disease (PD) is a common neurodegenerative disease characterized by the formation of Lewy bodies and the degeneration of dopaminergic neurons in the dense parts of the substantia nigra (SN) and striatum^1^. At present, the pathogenesis of PD remains unclear. Nonetheless, mitochondrial dysfunction is closely associated with protein quality control (PQC)^2^, and PQC disorder plays a very important role in the pathogenesis of PD^3^. Mitochondrial protein quality control (mtPQC) is the principal mechanism that maintainscell homeostasis. Among the pathways triggered by stress, the mitochondrial unfolded protein response (mtUPR) acts to restore proteostasis specifically within the mitochondria. The mtUPR reacts to mitochondrial proteotoxic stresses, such as the accumulation of unfolded or misfolded proteins, leading to upregulated expression of mitochondrial molecular chaperones (such as the heat shock proteins (HSPs) HSPE1, HSP60 and HSPA9) and proteases (such as CLPP, Lon, YME1L1, afg3l2 and SPG7) encoded by nuclear genes^4–7^. The mtUPR assists newly synthesized proteins folding correctly and it helps in the repair of misfolded or aggregated proteins, and the proteolytic removal of irreversibly damaged protein. Thus, the mtUPR is a mitochondrial-to-nuclear retrograde signal transduction pathway^8^ that ensures the quality of the mitochondrial proteome.

Many factors and various compounds can induce the mtUPR. Paraquat (an herbicide) and rotenone (a pesticide) are common toxins that trigger the mammalian mtUPR by inducing mitochondrial dysfunction^9–11^, both of which cause the production of oxidatively-damaged proteins^12^. The dopaminergic neurotoxin MPP^**+**^ is a structural analog of paraquat. It induces neurotoxicity by inhibiting complex I of the mitochondrial electron transport chain, leading to ATP depletion and oxidative stress^13^ that activates the mtUPR. Various experimental *in vivo* and *in vitro* models of PD have been developed by treatment with MPP^**+**^ or MPTP (an MPP^+^ precursor, 1-methyl-4-phenyl-1, 2, 3, 6-tetrahydropyridine)^14^.

**Figure 1:**
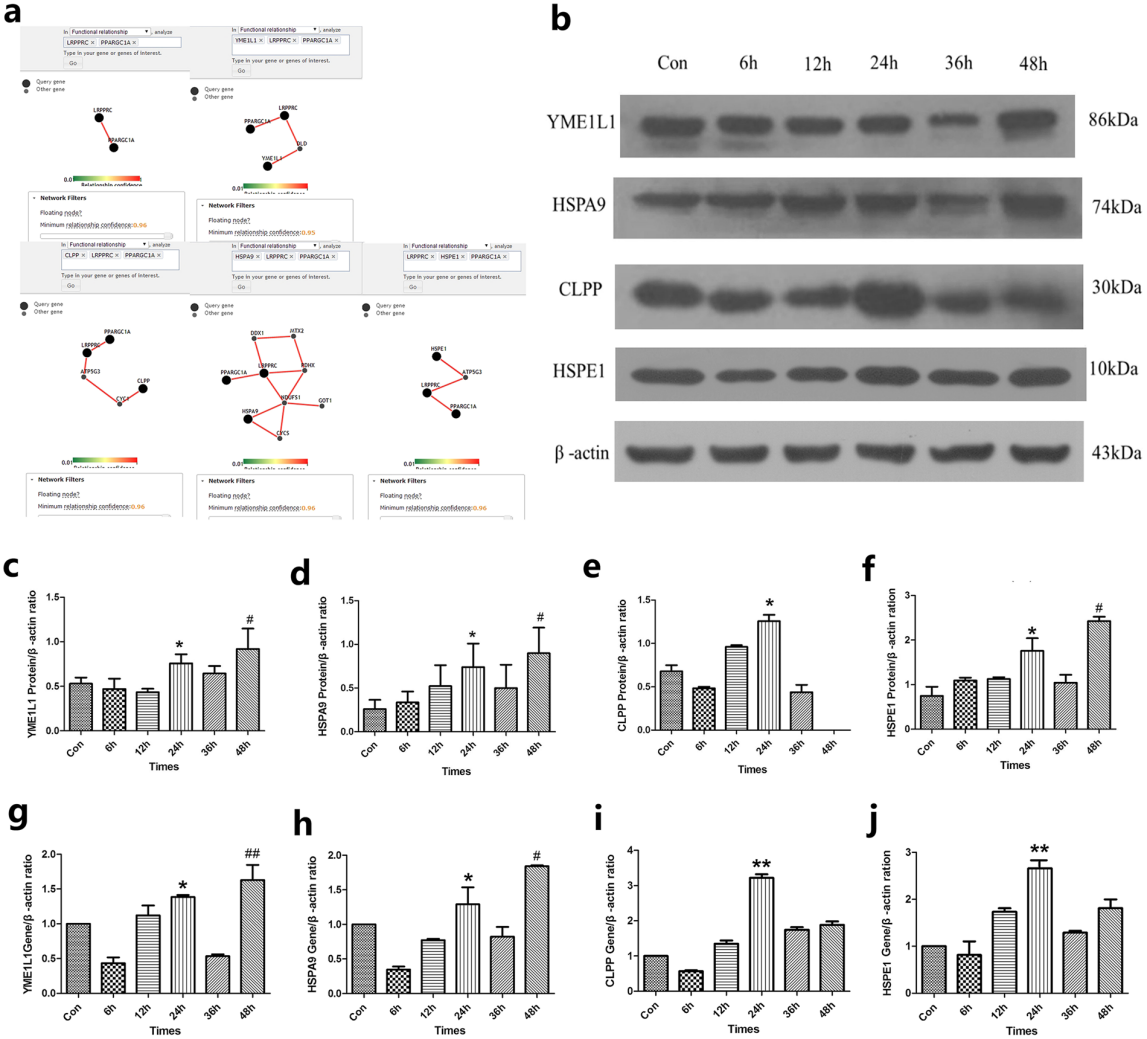
The expression pattern (proteins and mRNA) of YME1L1, HSPA9, CLPP and HSPE1 in response to MPP^+^ treatment. (**a**) Relation of PGC-1α/LRPPRC pathway and the chaperones and proteases. (**b**–**f**) Changes in the chaperones and protease proteins were observed at various time points following cell treatment with 1000 µM MPP^+^. (**b**) The expression of YME1L1, HSPA9, CLPP and HSPE1 proteins at various time points was detected by immunoblotting, full-length blots/gels are presented in Supplementary Fig. 2. (**c–f**) The expression of YME1L1, HSPA9, CLPP and HSPE1 protein at various time points was detected by Western blotting. (**c**) *P < 0.05, compared with Con, 6 h and 12 h; ^#^P < 0.05 compared with Con, 6 h and 12 h. (**d**) *P < 0.05, compared with Con and 6 h; ^#^P < 0.05, compared with all other groups, except 24 h. (**e**) *P < 0.05, compared with Con, 6 h, 12 h and 36 h. (**f**) *P < 0.05, compared with Con, 6 h, 12 h and 36 h ^#^P < 0.05, compared with all other groups. n = 8 for Western blots. (**g,h,i**) and (**j**) Changes in mRNA levels of chaperones and proteases following treatment with MPP^+^ 1000 μM at each time point. (**g**) *P < 0.05, compared with 6 h and 36 h; ^##^P < 0.01, compared with Con, 6 h and 36 h. (**h**) *P < 0.05, compared with 6 h, 12 h and 36 h; #P < 0.05, compared with all the other groups, except 24 h. (**i**) **P < 0.01, compared with all the other groups. (**j**) **P < 0.01, compared with Con, 6 h, 36 h and 48 h. n = 5 for real-time PCR. Note: Con (control group), 6 h (6 h group), 12 h (12 h group), 24 h (24 h group), 36 h (36 h group), 48 h (48 h group). All the data were analyzed by ANOVA, followed by Tukey’s LSD post hoc tests.

Peroxisome proliferator-activated receptor gamma (PPARγ) coactivator 1α (PGC-1α) plays an important role in mitochondrial biological processes and oxidative stress. It is a potent transcriptional regulator that is mainly expressed in tissues with high energy needs tahat are rich in mitochondria. When oxidative stress occurs, PGC-1α primarily aggregates in the nucleus^15^. Our last study showed that PGC-1α promoted mitochondrial transcription in both *in vivo* and *in vitro* models of PD; thus, protecting the dopaminergic neuronal-like cells in a model of PD^16,17^. The LRPPRC (leucine-rich pentatricopeptide repeat-containing) protein is a key member of the PPR (pentatricopeptide repeat motif) protein family, and it plays a major role in RNA processing, splicing, editing, stability and RNA translation initiation^18^. A mutation in the LRPPRC gene leads to the hereditary neurometabolic disorder French–Canadian subacute necrotizing encephalopathy, which is characterized by the lack of mitochondrial oxidative phosphorylation complex IV^19^. LRPPRC has been shown to interact with coactivator PGC-1α in the nucleus and regulate mitochondrial biosynthesis of nuclear gene expression^20^. Other literature studies indicate that LRPPRC plays an important role in the regulation of mtDNA expression posttranscription^21,22^. According to the PathwayNet tool (http://pathwaynet.princeton.edu/; Troyanskaya Laboratory, Princeton University, NJ, USA), we found the PGC-1α/LRPPRC pathway is closely related to mtUPR (Fig. 1a; where 0 is completely irrelevant and 1 is completely related). The diagram shows that PGC-1α/LRPPRC is related to YME1L1, CLPP, HSPA9 and HSPE1 by up to 0.95, 0.96, 0.96 and 0.96, respectively. Therefore, in mitochondrial stress, with the induction of mtUPR, we consider that PGC-1α might promote the synthesis of mtUPR-related proteases and molecular chaperones through the PGC-1α/LRPPRC pathway.

An adenovirus (Ad) vector is a noncoated, linear, double-stranded DNA virus that can be replicated independently in infected hosts. Due to its stability, broad range of hosts, high gene introduction rate, easy preparation and low pathogenicity in the human body, adenoviruses have become commonly used as highly versatile expression vectors^23^. Adenovirus vector systems, which have been widely used in fundamental and clinical research^24^, can specifically block or enhance the expression of related genes and proteins. SH-SY5Y cells, the third successive sub-clone of the metastatic myeloma SK-N-SH line, express tyrosine hydroxylase, dopamine-β-hydroxylase and the dopamine transporter in patients with neuroblastoma, and these cells have been widely used to establish a cell model for the study of the pathogenesis of PD. MPP^**+**^-induced SH-SY5Y cells are one of the best *in vitro* models of PD.

In this study, SH-SY5Y cells were intervened with MPP^+^ at various time points, to identify the optimal time that induced the mtUPR. SH-SY5Y cells were infected by adenovirus carrying PGC-1α (the target gene), resulting in overexpression of PGC-1α. Based on the close relationship between the PGC-1α/LRPPRC pathway by PathwayNet (Troyanskaya Laboratory, Princeton University) and mtUPR, the effect of PGC-1α overexpression on MPP^+^-induced mtUPR in SH-SY5Y cells and the effect of PGC-1α/LRPPRC signal transduction on mtUPR are discussed.

## Results

### After MPP**+** treatment, YME1L1, HSPA9, CLPP and HSPE1 protein expression was higher in the 24 and 48 h groups than in the other groups

The expression level of the molecular chaperones and proteases in SH-SY5Y cells was observed at the protein level after MPP^+^ treatment, and the activation of mtUPR was further observed under stress. Several observations were made following analysis of the results (Fig. 1b). (1) YME1L1 protein expression levels were increased after MPP^+^ treatment, 24 h (P < 0.05), and levels were 0.4, 0.6 and 0.7 times higher than they were in the non-MPP^+^ treatmentgroup and the 6 and 12 h groups, respectively levels were not significantly different (P > 0.05) from those in the 36 and 48 h groups. Likewise, the results from the 36 h group were not significantly different from those in non-MPP^+^ treatment, and 6 and 12 h groups. The 48 h treatment group had YME1L1 protein expression levels that were higher (P < 0.05) by 0.7, 0.9 and 1.1 times, respectively, compared with the non-MPP^+^ treatment and the 6 and 12 h groups. There was no significant difference between the 48 h treatment group and the 36 h group (P > 0.05) (Fig. 1c). (2) Similarly, the protein expression level of HSPA9 in the 24 h group increased (P < 0.05) and was 1.9 timeshigher than the level in the non-MPP^+^ treatment group and the 6 h group. There were no significant differences among the 12, 36 and 48 h groups (P > 0.05). There was no statistical significance (P > 0.05) among the 6 h, 12 h and 36 h groupsor the non-MPP+ treatment groups. The 48 h group had levels that were 3.5, 2.6, 1.7 and 1.8 times higher (all P < 0.05) than the levels in the non-MPP^+^ treatment and the 6, 12 and 36 h groups, respectively (Fig. 1d). (3) CLPP protein expression in the 24 h group increased (P < 0.05) by 1.8, 2.5, 1.4 and 3.0 times compared with the expression in the non-MPP^+^ treatment group, and the 6, 12, 36 h groups, respectively, but no protein was detected at 48 h (Fig. 1e). (4) In the same way, the expression level of HSPE1 protein in the 24 h group was 2.4, 1.6, 1.6 and 1.7 times (all P < 0.05) higher than it was the MPP^+^ group, and 6, 12 and 36 h groups, respectivelyand the difference between the 24 h the 48 h groups was statistically significant (P < 0.05) (Fig. 1f). From the above data analysis, CLPP, YME1L1, HSPA9 and HSPE1 protein levels were higher in the 24 and 48 h groups than they were in the other groups. However, in the 48 h group, the cells were and floating in the culture medium, so there was no measurable activity, the cells in the 24 h group grew well, which was similar to what was observed in our previous study^16^. Therefore, we considered 24 h was the best time of treatment.

### After MPP**+** treatment, the YME1L1, HSPA9, CLPP and HSPE1 transcriptional levels in 24 h were significantly higher than they were in the other groups

At the transcriptional level (Fig. 1g–j), (1) after MPP^+^ treatment, YME1L1 mRNA at 24 h was 3.5 and 2.4 times higher (P < 0.01) than what was observed at 6 and 36 h, respectively. There was no significant difference (P > 0.05) between the group without MPP^+^ treatment and the 48 h group. YME1L1 mRNA in the 48 h treatment group increased (P < 0.01)by 0.6, 3.0 and 2.2 times compared with the non-MPP^+^ treatment group, 6 h group and the 36 h group, respectively. There was no significant difference between the 12 h group and the 48 h group (P > 0.05) (Fig. 1g). (2) The transcriptional level of HSPA9 at 24 h increased by 2.7 (P < 0.01), 0.7 (P < 0.05) and 0.6 times (P < 0.05) compared with the 6, 12 and 36 h groups, respectively, but no statistical differences werefound for the MPP^+^ treatment group (P > 0.05). The 48 h group was statistically different (P < 0.05) from all the other groups (Fig. 1h). (3) At the transcriptional level, CLPP levels in the 24 h group were increased by (P < 0.01) 3.2, 5.6, 2.3, 1.8 and 1.7 times relative to the non-MPP^+^ treatment, and the 6, 12, 36 and 48 h groups,respectivly (Fig. 1i). (4) Moreover, the transcriptional level of HSPE1 only increased by (P < 0.01) 1.7, 2.1 and 1.5 times in the 24 h group relative to the non-MPP^+^ treatment, and 6, 36 and 48 h groups, respectively, which was not statistically different from the 12 h group (P > 0.05) (Fig. 1j). In summary, the transcription levels of YME1L1 and HSPA9 were most consistent with the protein levels in the 24 h group compared with other groups, whereas, the CLPP and HSPE1 transcriptional levels were significantly higher than the other groups. Combined with the level of protein expression in each group, we determined that 24 h was the best treatment time.

### The best MOI value of adenovirus-infected SH-SY5Y cells was determined to be 100

**Figure 2:**
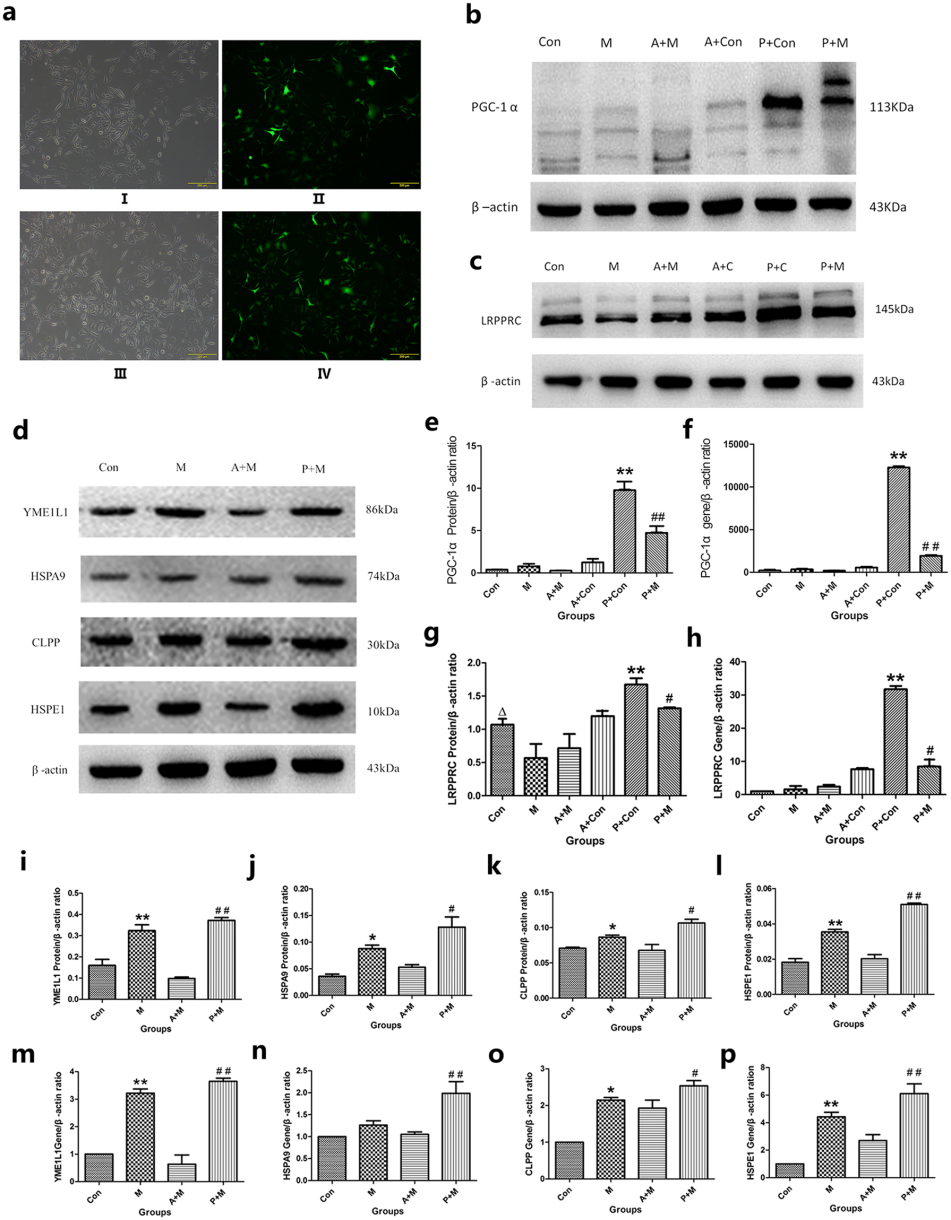
The overexpression of PGC-1α and its effect to the molecular chaperones and proteases of SH-SY5Y cells. (**a**) Fluorescence expression following treatment with the empty virus and the virus overexpression PGC-1α gene (Ad-GFP-PGC-1α) at MOI = 100. I,II,III and IVwere treated with empty virus control and Ad-GFP-PGC-1α virus respectively.I,III and II, V photographs were captured with light and green fluorescence microscopy, respectively. n = 4 for fluorescent images. (**b**,**e**,**f**) Comparison of immunoblots and mRNA transcriptional levels of PGC-1α in each group. (**b**,**e)** PGC-1α protein expression and PGC-1α protein expression profiles of each group. (**f)** The transcriptional level of PGC-1α protein was detected by real-time PCR. ^**^P < 0.01, compared with Con, Ad+Con and PGC-1α + MPP^+^; ^##^P < 0.01^,^ compared with Ad+MPP^+^. n = 5 for Western blots; n = 8 for real-time PCR analysis. Data were analyzed by ANOVA, followed by Tukey’s LSD post hoc tests. **(d**,**i**,**j**,**j**,**k**,**l)** Effect of *PGC-1α* overexpression on the protein expression levels of the molecular chaperones and proteases. Full-length blots/gels are presented in Supplementary Fig. 6. The samples were derived from the same experiment, and that gels/blots were processed in parallel. (**i**,**j**,**k**,**l)** molecular chaperones and proteases protein expression charts of each group. **i** ^**^P < 0.01, compared with Con; ^##^P < 0.01, compared with Ad+MPP^+^; (**j**,**k**,**l)** *P < 0.05, compared with Con; ^#^P < 0^.^05, compared with MPP + and Ad+MPP^+^. n = 6 for Western blots. (**m**–**p)** Effect of PGC-1α overexpression on the mRNA levels of chaperones and protease proteins. (**m)** ^**^P < 0.01, compared with Con; ^##^P < 0.01, compared with Ad+MPP^+^; (**n)** ^##^P < 0.01, compared with MPP^+^ and Ad+MPP^+^; (**o)** ^*^P < 0.05, compared with Con; ^#^P < 0.05, compared with Ad+MPP^+^; (**p)** ^**^P < 0.01, compared with Con; ^##^P^<^0.01^,^ compared with MPP^+^ and Ad+MPP^+^. n = 8 for real-time PCR analysis. (**c**,**g**,**h)** Effect of PGC-1α overexpression on LRPPRC expression. (**c**) Western blotting expression profiles of LRPPRC in each group, full-length blots/gels are presented in Supplementary Fig. 8. The samples were derived from the same experiment, and that gels/blots were processed in parallel. (**g)** LRPPRC protein expression profile; (**h)** Real-time PCR detected the transcriptional level of *LRPPRC*.^△^P < 0.05, compared with MPP^+^; ^**^P < 0.01, compared with Con and Ad+Con; ^#^P < 0.05 compared with MPP^+^, Ad+MPP^+^ and *PGC-1α* + Con. n = 6 for Western blots; n = 9 for real-time PCR analysis^.^ Note: Con (control group), M (MPP^+^ group), A + M (Ad+MPP^+^ group), A + Con (Ad+control group), P + Con (PGC^-^1α + control group), P + M (PGC-1α + MPP^+^ group). All the data were analyzed by ANOVA followed by Tukey’s LSD post hoc tests.

According to the early grouping analysis, in the case of empty Ad virus the PGC-1α gene overexpressing virus(Ad-GFP-PGC-1α), when the MOIs were all 100, infected SH-SY5Y cells were observed at 24 h, the cell infection rate was>90%, and cell death was low (<10%) (Fig. 2a). Therefore, an MOI = 100 was used in the next experiment.

### Establishing a PGC-1α overexpression dopaminergic neuronal-like cell model

Overexpression of the target gene PGC-1α was observed at the protein and mRNA levelsafter SH-SY5Y cells were infected by an adenovirus, to establiah a cell model. Regarding PGC-1α at the protein level, the results (Fig. 2b) indicated the PGC-1α overexpression group had significantly higher protein levels (increased (P < 0.01) by 27 and 17 times) than the cells without MPP^+^ treatment and the cells in the empty Ad virus treatment group, respectively. The overexpression of PGC-1α in the MPP^+^ treatment group was higher (P < 0.01) than it was in the MPP^+^ group (8-fold) and the empty virus plus MPP^+^ treatment group (19-fold) (Fig. 2e). Similarly, for PGC-1α at the transcriptional level (Fig. 2f), the mRNA level in the PGC-1α overexpression group remained markedly higher (P < 0.01) than it did in the non-MPP^+^ treatment group (65 times) and the empty virus treatment group (25 times). At the mRNA level, overexpression of PGC-1α in the MPP^+^-treated group was increased (P < 0.01) by 5.6 and 9.9 times compared with the levels in the MPP^+^ group and the empty virus plus MPP^+^ treatment group, respectively. The mRNA overexpression of PGC-1α in the MPP^+^-treated group was also 6.3 times lower (P < 0.01) than it was in the PGC-1α overexpression group. All the above data indicated that we successfully established a PGC-1α overexpression dopaminergic neuronal-like cell model.

### The expression of YME1L1, HSPA9, CLPP and HSPE1 was largely consistent with PGC-1α expression, as shown by Western blotting

Western blotting showed that YME1L1, HSPA9, CLPP and HSPE1 had molecular weights of 86, 74, 30 and 10 kDa, respectively (Fig. 2d). Full-length blots/gels are presented in Supplementary Fig. 5. The samples were derived from the same experiment and the gels/blots were processed in parallel. Analysis using GraphPad Prism 5 software revealed several findings. (1) After MPP^+^ treatment, the level of YME1L1 was increased by 1.1 times over that of the non-MPP^+^ treatment group (P < 0.01), PGC-1α overexpression by the MPP^+^ treatment group was 2.8 times higher than that of the empty virus group with MPP^+^ treatment (P < 0.01), and no significant difference was found between the MPP^+^ treatment groups (Fig. 2i). (2) Similarly, after MPP^+^ treatment, the level of HSPA9 increased by 1.4 times that of over the non-MPP^+^ treatment group (P < 0.05), and the levels in the the PGC-1α overexpression plus MPP^+^ group were 0.45 (P < 0.05) and 2.4 times (P < 0.01) higher than that of MPP^+^ treatment group and empty virus via MPP^+^ treatment group, respectively (Fig. 2j). (3) After MPP^+^ treatment, the expression level of CLPP was not significantly different from the control group (P > 0.05). In the overexpression group treated with MPP^+^, CLPP levels increased (P < 0.05) by 0.57 and 2.4 times over those of the MPP^+^ group and the empty virus plus MPP^+^ treatment group, respectively (Fig. 2k). (4) For the HSPE1 protein, the MPP^+^ treatment group increased levels by 94% (P < 0.01) compared with the control group; the PGC-1α overexpression plus the MPP^+^ treatment group increased (P < 0.01) levels by 46% and 1.6 times, respectively, compared to the MPP^+^ group and the empty virus plus MPP^+^ treatment group (Fig. 2l). The expression of YME1L1, HSPA9, CLPP and HSPE1 was largely consistent with the expression of PGC-1α.

### Overexpression of PGC-1α increased the expression of YME1L1, HSPA9, CLPP and HSPE1, as shown by real-time PCR

At the transcriptional level (Fig. 7), (1) after MPP^+^ treatment, YME1L1 levels increased 2.2-fold over the nontreatment group (P < 0.01). The overexpression of PGC-1α was 4.6 times higher than that of the MPP^+^-treated group (P < 0.01), and the levels were statistically similar to that of the MPP^+^ group (P > 0.05) (Fig. 2m). (2) Similarly, the PGC-1α overexpression in the MPP^+^ treatment group increased by 0.57 and 0.97 times in comparison to the MPP^+^ group and the empty virus with MPP^+^ treatment group, respectively (P < 0.05; P < 0.01), and the HSPA9 level of the nontreated group was statistically comparable to the levels after MPP^+^ treatment (Fig. 2n). (3) After MPP^+^ treatment, the CLPP protein levels increased by 1.1-fold over the levels of the control group (P < 0.05). In the PGC-1α overexpression group, the MPP^+^ treatment group increased CLPP protein levels by 0.34 times (P < 0.05) relative to the empty virus plus MPP^+^ treatment group, but there was no significant difference compared with the MPP^+^ group (P > 0.05) (Fig. 2o). (4) For the HSPE1 protein, the MPP^+^ treatment group increased levels by 3.4 times compared to the levels of the control group (P < 0.01). In the group with PGC-1α overexpression and MPP^+^ treatment, HSPE1 levels increased (P < 0.01) by 0.45 and 1.6 times over those of the MPP^+^ group and the empty virus via MPP^+^ treatment, respectively (Fig. 2p). In summary, the expression trend of YME1L1, HSPA9, CLPP and HSPE1 was approximately consistent with PGC-1α, indicating that the overexpression of PGC-1α increased the expression of related molecules in the mtUPR.

### PGC-1α overexpression promotes the expression of LRPPRC protein

LRPPRC has a molecular weight of 130 kDa (Fig. 2c,g). Using GraphPad Prism 5 image analysis software, the level of LRPPRC after MPP^+^ treatment was found to be 2.4 times (P < 0.05) lower than it was in the non-treatment group. In the PGC-1α overexpression group (without MPP^+^ treatment), LRPPRC increased by 0.56 and 0.49 times in comparison to the control group and empty virus without MPP^+^ treatment group, respectively (P < 0.01; P < 0.05). For LRPPRC in the PGC-1α overexpression group, the expression level decreased by 26% (P < 0.05) after MPP^+^ treatment, but levels were 1.35 times higher (P < 0.05) than they were in the MPP^+^ group without overexpression of PGC-1α;further, LRPPRC levels were increased by 84% (P < 0.05) compared with the empty virus plus MPP^+^ treatment group. PGC-1α overexpression promotes the expression of LRPPRC protein, while treatment with MPP^+^ reduces the expression of LRPPRC protein.These results are similar to what was obserbed with PGC-1α following MPP^+^ treatment, which strongly suggests a correlation between LRPPRC protein and PGC-1α.

### PGC-1α overexpression promotes the expression of LRPPRC mRNA

At the transcriptional level (Fig. 2h), the level of LRPPRC after MPP^+^ treatment was statistically comparable to that of the control group (P > 0.05). For the PGC-1α overexpression group (without MPP^+^), LRPPRC was 31 and 4.2 times higher (P < 0.01; P < 0.01), than it was in the MPP^+^ untreated group and the PGC-1α overexpression group. In the PGC-1α overexpression groups, LRPPRC was 2.8 times lower in the MPP^+^ treatment group than it was in the non-MPP^+^ treatment group (P < 0.05), but 5.3 times higher than it was in the MPP^+^ group without PGC-1α overexpression (P < 0.05); futher, LRPPRC level were 3.5 times higher than they were in the empty virus group with MPP^+^ treatment (P < 0.05). The LRPPRC protein level appears to be the same as its RNA level. We believe that PGC-1α overexpression promotes the expression of LRPPRC and PGC-1α may be an upstream activator of LRPPRC.

### LRPPRC may be transferred from the nucleus to the cytoplasm after treatment with MPP**+** and PGC-1α overexpression

**Figure 3:**
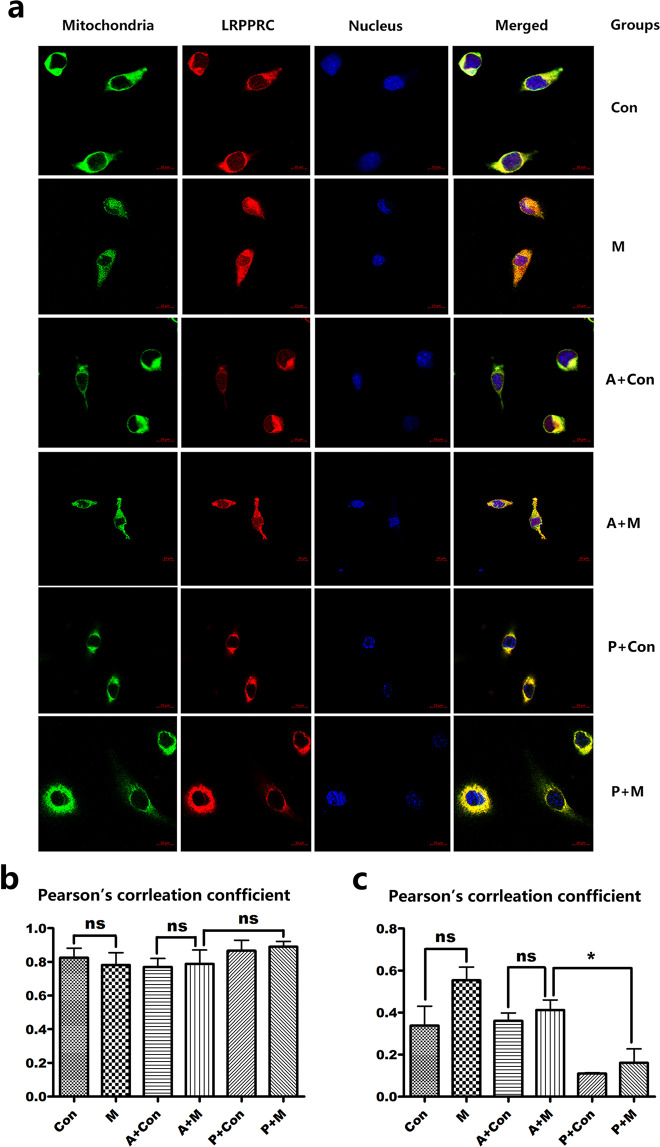
Subcellular localization of *LRPPRC* following of PGC-1α overexpression. n = 4; visulization was performed using fluorescence confocal microscopy. (**a**) The immunofluorescence results of LRPPRC subcellular localization. Mitochondria for 647 channels of excitation light, appear in green; LRPPRC is 594 channels of excitation light, appearing in red; the nucleis are stained by DAPI and appear in blue. Note: Con (control group), M (MPP^+^ group), A + M (Ad+MPP^+^ group), A + Con (Ad+control group), P + Con (PGC-1α + control group), P + M (PGC-1α + MPP^+^ group). (**b**,**c)** The Pearson’s corrleation confficient of LRPPRC in mitochondria and nucleus. ns, no statistical significance; *P < 0.05 PGC-1α + MPP^+^ compared with MPP^+^.

Cell immunofluorescence results (Fig. 3) showed that under normal circumstances, LRPPRC was mainly distributed in mitochondria, with obvious co-location with mitochondria. There was a little floccus in the nucleus, with a small amount. After MPP^+^ intervention, LRPPRC was mainly distributed in mitochondria, and its co-localization was obvious, which in the nucleus increased slightly, showing a granular floccus. LRPPRC appeared to exit the nucleus, which in the nucleus increased slightly, showing a granular floccus. After overexpression of PGC-1α, LRPPRC was mainly distributed in mitochondria with obvious co-localization, and almost no LRPPRC floccus in the nucleus.. This phenomenon was most obvious in the group without MPP^+^ treatment. These results suggested that most of the LRPPRC is located on mitochondria whether or not it is interfered with MPP^+^ or overexpression of PGC-1α, and there is no obvious metastasis. After the addition of MPP^+^, LRPPRC could be transferred to the nucleus slightly, but there was no significant statistical difference. After overexpression of PGC-1α, LRPPRC showed nuclear transferred from the nucleus and colocalization decreased in the nucleus, with a statistical difference (P < 0.05). In summary, LRPPRC is mainly distributed in mitochondria, and is partly located in the nucleus of SH-SY5Y cells.Under the intervention of MPP^+^, a small amount of LRPPRC can be transferred to the nucleu, but there was no statistical difference (P = 0.08) (Fig. 3b). After overexpression of PGC-1α (with the intervention of MPP^+^), it was found that the expression of LRPPRC was transferred to the outside of the nucleus, and the colocalization with the nucleus was reduced, and the difference was statistically significant (P < 0.05) (Fig. 3c).

## Discussion

PD is a prevalent neurodegenerative disease in the middle-aged and the elderly population, with serious impacts on the physical and mental health. The patients not only present dyskinesia symptoms but at the advanced stage, PD is also associated with cognitive impairment, impaired memory function, anxiety and depression^25–27^. Mithchondria participate in the generation of energy, regulate the storage of calcium ions, contribute to signal transduction in cells and induce apoptosis, which is of great significance for the prevention and treatment of PD^28^.In PD patients, the activity of the mitochondrial electron transport chain complex I in the SN decreases^29,30^. This is closely related to MPTP (methylphenyltetrahydropyridine, MPTP) and MPP^+^, in which can lead to the loss of dopaminergic neurons^31–33^ predominantly through the production of ROS. These process can promote the mtUPR^12^.

The mtUPR is an emerging and adaptive stress response pathway that ensures the best quality and function of the mitochondrial proteome and interacts with other mitochondrial mass control systems, such as oxidative stress reaction, mitochondrial biosynthesis, mitochondrial autophagy and the ubiquitin protease system, which maintain the normal mitochondrial structure and function^34–37^. The mtUPR internally monitors mitochondrial protein homeostasis and respond to stress signals, and it activates the complex mtPQC network^38^. Dopaminergic neurons contain a relatively low mass of mitochondria^39^, and the mitochondria with complete functional proteins are the key^40^ to guarantee the free radical scavenging mechanism. The mtUPR molecular chaperones and proteases are discussed further in the following sections. According to our literature review and previous study^16^, when the concentration of MPP^+^ is 1000 µM, the cell death rate is only 22.38% (P < 0.05), making it the most suitable for cell modeling. Next, we continued to observe the changes in the levels of the proteases (YME1L1 and CLPP) and chaperones (HSPA9 and HSPE1) at various time points. We found that at the protein level, both the 24 and 48 h groups showed significant differences in the molecular chaperones (HSPA9 and HSPE1) and proteases (YME1L1 and CLPP) (Fig. 1b). Among the time points evaluated, the transcriptional levels of CLPP and HSPE1 were significantly higher at 24 h, and YME1L1 and HSPA9 were significantly higher at 24 and 48 h (Fig. 1g–j). As the cells were dead at 48 h, most could not attached to the well and floated in the medium because when mtUPR exceeds its protective ability, the cells inevitably undergo lysis, autophagy and apoptosis^41,42^. Consequently, we chose 24 h, as the best intervention time.

PGC-1α is a potent transcriptional cofactor encoded by the *PPARGC1A* gene and plays a vital role in mitochondrial biosynthesis and oxidative phosphorylation^43,44^. The mtUPR can hinder aging, but the relationship between PGC-1α overexpression and the mtUPR is not yet fully understood. Therefore, we overexpressed PGC-1α by an adenovirus vector to study the association. Similar to previous studies, when the MOI value was 100, the rate of adenovirus infection (more than 90%) and the mortality rate (10%) were most suitable for cell modeling (Fig. 2a)^16^. As expected, the PGC-1α overexpression group had higher protein and mRNA levels of PGC-1α than the other groups (Fig. 2b,e,f). MPP^+^ treatment of SH-SY5Y cells overexpressing PGC-1αdecreased the protein and mRNA levels of PGC-1α by 1.6 and 6.3 times, respectively (Fig. 2e,f), which was consistent with previous studies^16^ and suggested that we had established a successful model.

The hallmark of mitochondrial retrograde signaling is the alteration of the expression of nuclear genes elicited by a signal from the mitochondria (mainly proteases and molecular chaperones), which alerts the cell of perturbations in mitochondrial homeostasis^45^. YME1L1 is a member of the AAA family of ATPases, affecting the morphology and function of mitochondria^46,47^. Assisted by the mitochondrial processing peptidase, YME1L1 removes its mitochondrial targeting sequence, then enters the mitochondria^48^. In this experiment, the levels of YME1L1 protein and mRNA were increased by overexpression of PGC-1α (Fig. 2i,m). We speculate whether the YME1L1 transcription mRNA encoded by the nuclear genome is somehow increased by PGC-1α and is transported to the mitochondria for extensive expression, which remains to be further confirmed. The mitochondrial matrix protease Clp family consists of ClpAP, ClpCP, ClpEP, ClpXP and ClpYQ, which exist in the mitochondria of various prokaryotes and eukaryotes, as well as in the chloroplasts of algae and plant cells^49^. The active two-chain proteases are complex compounds consisting of a peptidase subunit (CLPP or ClpQ). Clp maintains the stability of mitochondrial proteins by degrading oxidatively damaged proteins^50,51^. Following the appropriate stimulation of MPP^+^, ROS leads to increased protein oxidation, and the induced CLPP expression and transcription validated the treatment parameters (Fig. 2k,o). In the PGC-1α overexpression group, CLPP was increased by promoting mRNA splicing of CLPP precursors (Fig. 2o)^18,52^. The interrupted proteostasis is closely linked to age-related diseases.

Unfolded and misfolded proteins in the mitochondria are mainly recovered by HSPA9, HSP60 and HSPE1. HSPA9 mediates the essential functions of mitochondrial protein import and synthesis^53^. The level of HSPA9 decreased in PD patients’ SN, suggesting that mtUPR is involved in the pathophysiological mechanism of PD^54^. In this experiment, we found the expression of HSPA9 increased after PGC-1α overexpression (Fig. 2j,n), indicating that HSPA9 may not only participate in mtUPR but also play a role in autophagy. Human HSP10 protein is encoded by the nuclear HSPE1 gene; HSPA9, HSP60 and HSPE1 form a complex involved in mitochondrial protein folding, which is closely related to PGC-1α biosynthesis^55,56^, but we have not found strong evidence for a correlation between HSPE1 and PGC-1α. Although we observed an increase in the level of HSPE1 expression (Fig. 2l,p), we still need to explore the relationship further.

LRPPRC participates in mitochondrial RNA metabolism, and it regulates some mitochondrial encoding genes and other similar genes with PGC-1α to form the PGC-1α/LRPPRC signal pathway^57^. After LRPPRC deletion, PGC-1α co-activation expression ability is significantly reduced^58^. In mitochondria, LRPPRC interacts with the translation initiation factor 4E (eIF4E) to regulate the export, stability and translocation of mRNA^59^ following its transcription. Studies have shown that in aging rats, the formation of PGC-1α/LRPPRC complex, which is induced by aerobic exercise and inhibition of the FoxO pathway, can trigger atrophic skeletal muscle regeneration^20^. We observed the effect of LRPPRC on PGC-1α overexpression. After MPP^+^ treatment, the level of LRPPRC protein was 2.4 times lower than that in the non-MPP^+^ treatment group. In the PGC-1α overexpression without MPP^+^ group, the LRPPRC level increased by 56% (P < 0.01) and 49% (P < 0.05), respectively, compared to the empty virus plus MPP^+^ group and the empty virus without MPP^+^ group, which was similar to the PGC-1α treatment group. In PGC-1α overexpression after the MPP^+^ treatment group, the LRPPRC expression level reduced by 26% relative to the levels in the non-MPP^+^ group (Fig. 2g). Similar results were observed in LRPPRC, at the transcriptional level (Fig. 2h).

These findings suggest that the PGC-1α/LRPPRC complex is damaged after MPP^+^ treatment, resulting a decrease in PGC-1α/LRPPRC protein expression. We also found that after MPP^+^ treatment with overexpression of PGC-1α, the fluorescent LRPPRC signal in the nucleus was reduced (Fig. 3). This phenomenon was most evident in the non-MPP^+^ treatment group with PGC-1α overexpression, indicating that some LRPPRC may have been transferred from the nucleus to the cytoplasm. We speculate the PGC-1α/LRPPRC complex can reduce the damage of mitochondrial oxidative stress by inhibiting the ubiquitin/proteasome system. Knocking out LRPPRC in mammalian cells leads to the imbalance of the complex IV subunit of the mitochondrial and nuclear co-coding complex, which triggers the mtUPR^60^. Thus, we believe that PGC-1α/LRPPRC largely regulates mtUPR. This observation may support the involvement of LRPPRC in the pathogenesis of PD, but whether the LRPPRC protein is reduced in the PD brain remains to be further studied.

In summary, our results show that the oxidative damage caused by MPP^+^ in the PD cell model can induce the early activation of the mtUPR pathway, which increases the expression of the molecular chaperones and proteases regulated by the reaction. When mtUPR is activated, the expression of glycolytic gene and some amino acid genes is upregulated. As a powerful transcription factor, PGC-1α plays a role in energy homeostasis and interaction of the mitochondrial genes, such as mitochondrial transcription factor A (TFAM), estrogen-related receptor alpha (ERRα), and nuclear respiratory factor-1 (NRF-1) and -2 (NRF-2)^16,61^. However, what is the relationship between mtUPR and these genes? Based on the close relationship between PGC-1α/LRPPRC pathway and mtUPR depicted by PathwayNet (Troyanskaya Laboratory, Princeton University), we explored this association. The results reveal that the expression of PGC-1α may inhibit the oxidative damage caused by MPP^+^ by increasing the expression of molecular chaperone and protease genes in the mtUPR pathway. The PGC-1α/LRPPRC signaling pathway is involved in the specific mechanism, we need to explore further. Accordingly, we speculate that PGC-1α/LRPPRC signal transduction may maintain mitochondrial homeostasis through mtUPR. It is possible that mtUPR plays a role in slowing down the progress of PD.

## Materials and methods

### Cell culture and MPP^+^ treatment

Human SH-SY5Y neuroblastoma cells were obtained from the Chinese Academy of Sciences Committee Type Culture Collection cell bank and were cultured in Dulbecco’s modified Eagle’s medium (DMEM/F12, HyClone, Logan, UT, USA) supplemented with 10% fetal bovine serum (Gibco, Grand Island, NY, USA), 100 U/ml penicillin (HyClone, Logan, UT, USA), and 100 U/ml streptomycin (HyClone, Logan, UT, USA) (complete media, CM). The cell line was cultured in 100 mm tissue culture plates at 37 °C in a humidified incubator (Model No. 3130, Forma Scientific, Ohio, USA) containing 5% CO_2_. When the cell density reached 80-90%, the cells were harvested and dispersed. We replaced the culture medium every 2 days. The cells in CM were treated with 1000 µM MPP^+^ (D048, Sigma-Aldrich, St. Louis, MO, USA) for various times (6, 12, 24, 36 and 48 h) the control cells were incubated with culture medium containing PBS. Thus, six experimental groups were defined. For the empty control group (Con), the SH-SY5Y cells were cultured for 72 h, which was followed by the addition of phosphate-buffered saline (PBS) and the cells incubated for 24 h before examination and analysis. For the five MPP^+^ treatment groups, after the SH-SY5Y cells were cultured for 72 h, complete medium with a final concentration of 1000 µM MPP^+^ was added, and the cells incubated for 6, 12, 24, 36 and 48 h, before detection and analyses were carried out.

### Viral infection

A human PGC-1α adenovirus was constructed, purified and amplified by SBO Medical Biotechnology Co. Ltd (Shanghai, China). The PGC-1α adenovirus contained the gene for GFP, in tandem with the PGC-1α gene. The optimal multiplicity of infection (MOI) was determined to be 100:1, based on the observed fluorescence intensity of GFP, the survival rate and the rate of infection in SH-SY5Y cells(from our previous study)^16^. Human SH-SY5Y cells were infected with adenovirus (MOI = 100) for X h (X represents the most effective time point). Six experimental groups were defined. (1) The negative control group (Con) was as follows: SH-SY5Y cells were cultured for 72 h, PBS was added, and the cells were incubated for *X* h before examination and analysis. (2) The MPP^+^ group (M) was as follows: after culture of SH-SY5Y cells for 72 h, a final concentration of 1000 µM MPP^+^ was added to the complete medium, and X h was determined. (3) The Ad + control group (A + Con) was as follows: for the empty Ad virus plus nontreatment group, SH-SY5Y cells were cultured for 24 h. Culture medium (without FBS) containing an empty Ad virus was then added, and the cells were cultured for 4 h, during which time, the culture plate/bottle was shaken every 15 min. After 48 h, the old medium was discarded; then 10% FBS DMEM/F12 medium was added, and the cells wereincubated for *X* h. After collecting the cells, they were analyzed. (4) The Ad+MPP^+^ group (A + M) was as follows: after 24 h of culture, SH-SY5Y cells were cultured with an empty Ad virus medium (without FBS) for 4 h. During this period, the culture plate/bottle was shaken every 15 min. After 48 h, the old medium was discarded and then a complete medium containing a final concentration of 1000 µM MPP^+^ was added to the cells for incubation. After *X* h, the cells were collected and analyzed. (5) The PGC-1α + control group (P + Con) was as follows: for the overexpression of Ad-GFP-PGC-1α plus nontreatment group, SH-SY5Y cells were cultured for 24 h. Next, the culture medium (without FBS) containing an adenovirus that overpresses Ad-GFP-PGC-1αwas added before culturing for 4 h, during which time, the culture plate/bottle was shaken every 15 min. After 48 h, the old medium was discarded and 10% FBS DMEM/F12 medium was added and incubated for *X* h. After collection the cells, they were analyzed. (6) The PGC-1α + MPP^+^ group (P + M) was as follows: for the overexpression of Ad-GFP-PGC-1α plus MPP^+^ treatment group, SH-SY5Y cells were cultured for 24 h, and then the culture medium containing the Ad-GFP-PGC-1α overpressing adenovirus was added and incubated for 4 h, during which time, the culture plate/bottle was shaken every 15 min. After 48 h, the old medium was discarded; then a complete medium containing a final concentration of 1000 µM MPP^+^ was added and incubated for *X* h. After collection, the cells were detected and analyzed.

### Quantitative real-time polymerase chain reaction (PCR) analysis

Total RNA from SH-SY5Y cells was isolated according to the manufacturer’s protocol using TRIZOL reagent (Invitrogen, Carlsbad, CA, USA). Total RNA purity and integrity were confirmed using an ND-1000 NanoDrop (NanoDrop Technologies) and a 2100 Bioanalyzer (Agilent). RNA (1 μg) was reverse-transcribed to cDNA in a total volume of 20 μl using a Revert Aid TM First Strand cDNA Synthesis Kit (Fermentas, St. Leon-Rot, Germany). The cDNA (2 μl) was amplified with an ABI prism 7500 HT sequence detection system (Applied Biosystems, Forster City, CA, USA) in a total volume of 20 μl containing 10 μl of the FastStart Universal SYBR Green Master Mix (ROX) (Roche, Germany). Forward and reverse primers for specific amplification were designed and are -in Table 1; they were designed to eliminate the possibility of amplifying genomic DNA. Quantitative real-time PCR was performed using an ABI Prism 7500 HT sequence detection system, based on the 59-nuclease assay for the various genes indicated and the housekeeping gene β-actin. Relative expression was calculated using the ΔΔCt method, once primers passed the validation experiment. The results are expressed as an average of triplicate samples of at least three independent experiments for control and treated cells.

**Table 1:** The sequence of forward and reverse primers.

Primer name	TargetSeq (5′to3′)
PGC-1α-F PGC-1α-R	F1(5′-TGCCACCACCATCAAAGAAGC-3′), R1(5′-TCACCAAACAGCCGCAGACT-3′)
LRPPRC-F LRPPRC-R	F1(5′-AGATGGCCCAAGTGTCTTTG-3′), R1(5′-AGGAAAGGAGTGCATCTGGA-3′)
YME1L1-F YME1L1-R	F1(5′-CCAGCAGTGAGCCTTCACTTA-3′), R1(5′-AACCCCGAGACTGTATGAAAACAT-3′)
HSPA9-F HSPA9-R	F1(5′-GGGTACTACCAACTCCTGCG-3′), R1(5′-GGCATTCCAACAAGTCGCTC-3′)
CLPP-F CLPP-R	F1(5′-CTCATTCCCATCGTGGTGGA-3′), R1(5′-GATAACAAGGCTGGCAACGC-3′)
HSPE1-F HSPE1-R	F1(5′-AGTAGTCGCTGTTGGATCGG-3′), R1(5′-GGTGCCTCCATATTCTGGGA-3'
β-actin-F β-actin-R	F1(5′-AGAAGGCTGGGGCTCATTTG-3′), R1(5′AGGGGCCATCCACAGTCTTC-3′)

### Western blot analysis

Cells were seeded into 75 cm^2^ culture flasks and treated as above. For whole cell lysates, the cells were washed twice with ice-cold PBS, harvested in RIPA Lysis Buffer [50 mM Tris pH 7.4, 150 mM NaCl, 1% Triton X-100, 1% sodium deoxycholate, 0.1% SDS, 1 mM sodium orthovanadate, 50 mM sodium fluoride, and 1 mM EDTA (Ethylenediaminetetraacetic acid)], incubated on ice for 10 min, and centrifuged at 12,000 × g for 10 min at 4 °C. Then the supernatant containing cell lysates was collected. Equal amounts of protein (45 μg) from the cell extracts of each treatment condition were separated using 8% sodium dodecyl sulfate-polyacrylamide gel electrophoresis, and then were transferred electrophoretically onto polyvinylidene fluoride membranes. The blots were blocked by incubation in 5% (w/v) nonfat dry milk in PBS with 0.1% Tween 20 (PBS-T), for 3 h. After incubation with primary antibodies (anti-CLPP 1:2000, anti-HSPA9 1:500, anti-YME1L1 1:1000, anti-HSPE1 1:10000, anti-β-actin 1:2000, anti-PGC-1α 1:1000) in PBS-T at 4 °C overnight, the membranes were washed three times in PBS-T for 10 min. Subsequently, the membranes were incubated for 1.5 h in PBS-T containing secondary antibody conjugated to horseradish peroxidase (anti-mouse IgG 1:2000 and anti-rabbit IgG 1:2000). The immunoreactive bands were visualized and quantified using a chemiluminescent Luminata^TM^ Forte Western HRP substrate. Protein levels were normalized to the housekeeping protein β-actin, to adjust for the protein loading variability, and expressed as a percentage of the vehicle control (deemed to be 100%).

### Cell immunofluorescence

SH-SY5Y cells seeded onto coverslips in 6-wells plates were incubated as described above. The cells were fixed and permeabilized with 4% paraformaldehyde and 0.1% Triton X-100, respectively. Cells were blocked with 1% normal donkey serum (Merck, Darmstadt, Germany) in PBS for 30 min at room temperature and then incubated with the primary antibody (LRPPRC 1:2000) diluted in PBS-T, at 4 °C overnight. Labeled donkey anti-mouse IgG (Cy3, red label, 1:1000), diluted in PBS-T, was used as the secondary antibody and was incubated with the cells in the dark for 2 h at room temperature. Then, the cells were incubated with 0.5 μg/mL 4′,6-diamidino-2-phenylindole (DAPI) at room temperature for 30 min and then they were mounted in anti-quenching medium. Finally, photomicrographs were obtained using confocal microscopy (Leica SP5).

### Statistical analysis

All quantitative data were collected from at least three independent experiments. The final data are expressed as the mean ± SEM, and analyzed using SPSS 17.0 statistical software (SPSS, Inc., Chicago, IL, USA) by means of one-way ANOVA, followed by Tukey’s multiple comparison post hoc test. Real time PCR data (Ct) were translated into the 2^−△△Ct^ format for statistical analysis; differences between mean values were analyzed by one-way analysis of variance (ANOVA), *P* < 0.05 and *P* < 0.01 were considered significant.

## Supplementary information

Supplementary Information.

## Data Availability

The datasets supporting the conclusions of this article are included within the article.
