# Supplementary material for: Overexpression of PGC-1α influences the mitochondrial unfolded protein response (mtUPR) induced by MPP+ in human SH-SY5Y neuroblastoma cells
Source: Sci Rep. 2020 Jun 26;10:10444. doi: 10.1038/s41598-020-67229-6 (PMC7320005; doi:10.1038/s41598-020-67229-6)

**Overexpression of PGC-1α influences the mitochondrial unfolded** **protein response (mtUPR) induced by MPP^+^ in human SH-SY5Y neuroblastoma cells**

Yousheng Cai^1,2,+^, Hui Shen^1,+^, Huidan Weng^1, 3^, Yingqing Wang^1^, Guoen Cai^1^, Xiaochun Chen^1, 3^, Qinyong Ye^1, 3*^

^1^Department of Neurology, Fujian Institute of Geriatrics, Fujian Medical University Union Hospital, 29 Xinquan Road, Fuzhou, Fujian 350001, China.

^2^ Department of Neurology, Zhangzhou Affiliated Hospital of Fujian Medical University, 59 Shengli Road, Zhangzhou 363000, China.

^3^ Institute of Neuroscience, Fujian Key Laboratory of Molecular Neurology, Fujian Medical University, 29 Xinquan Road, Fuzhou 350001, China.

*Corresponding author: [unionqyye@163.com](mailto:unionqyye@163.com).

^+^ Yousheng Cai and Hui Shen contributed equally to this work and should be considered co-first authors.

Email addresses:

Yousheng Cai: [746862048@qq.com](mailto:746862048@qq.com)

Hui Shen: [wsyj8983@sina.com](mailto:wsyj8983@sina.com)

Huidan Weng：whdmls@163.com

Yingqing Wang: [249087491@qq.com](mailto:249087491@qq.com)

Guoen Cai: [cgessmu@126.com](mailto:cgessmu@126.com)

Xiaochun Chen: [chenxc998@163.com](mailto:chenxc998@163.com)

Qinyong Ye: [unionqyye@163.com](mailto:unionqyye@163.com)


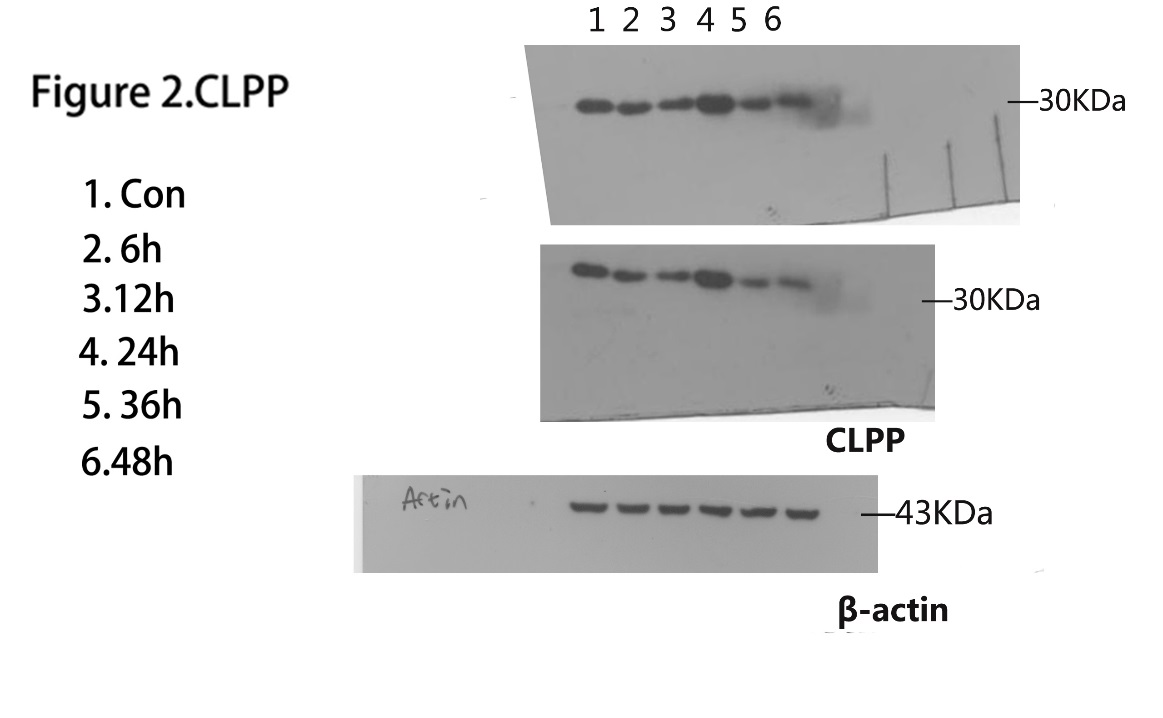


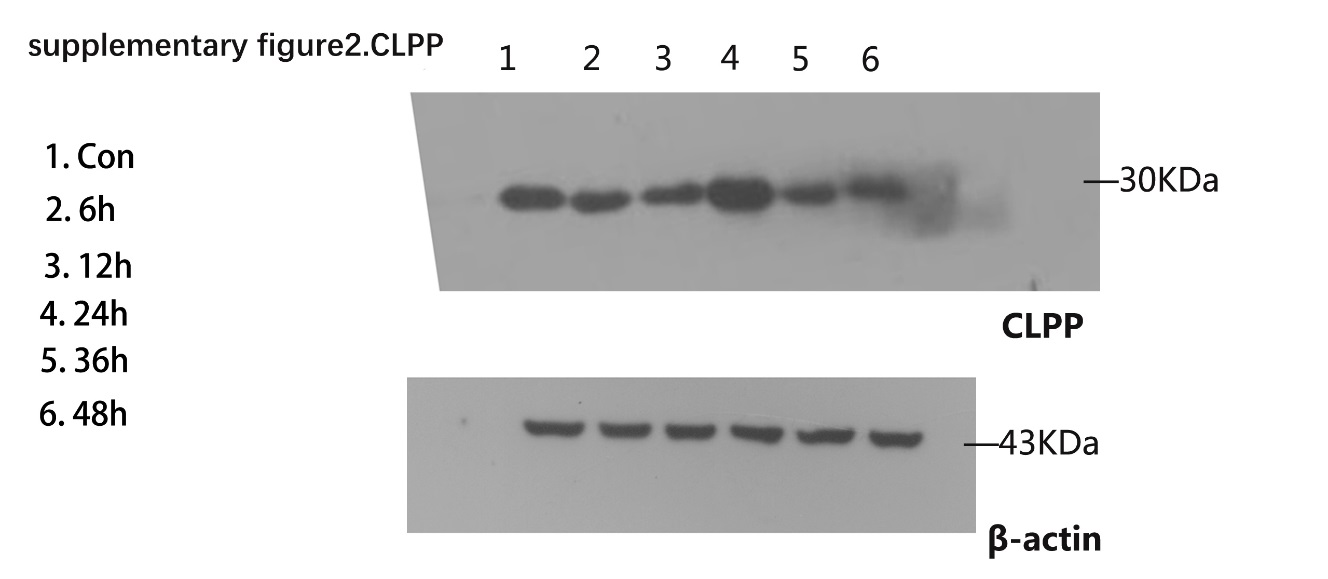


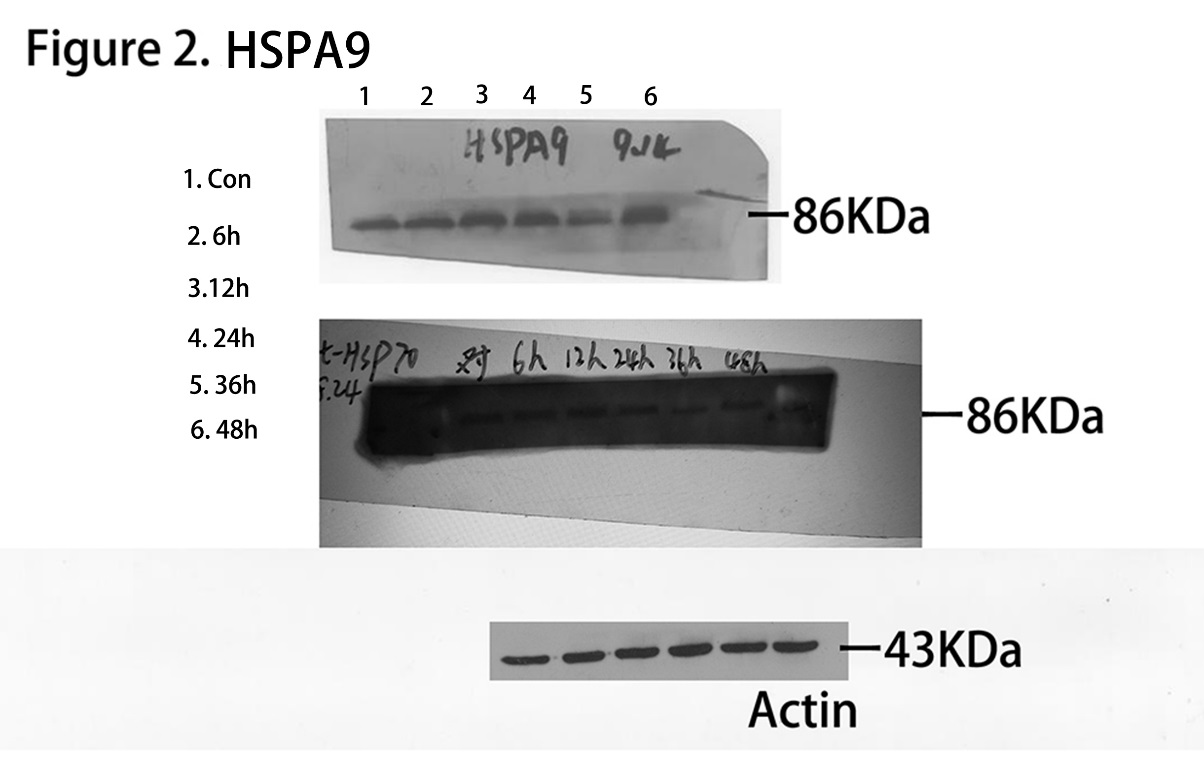


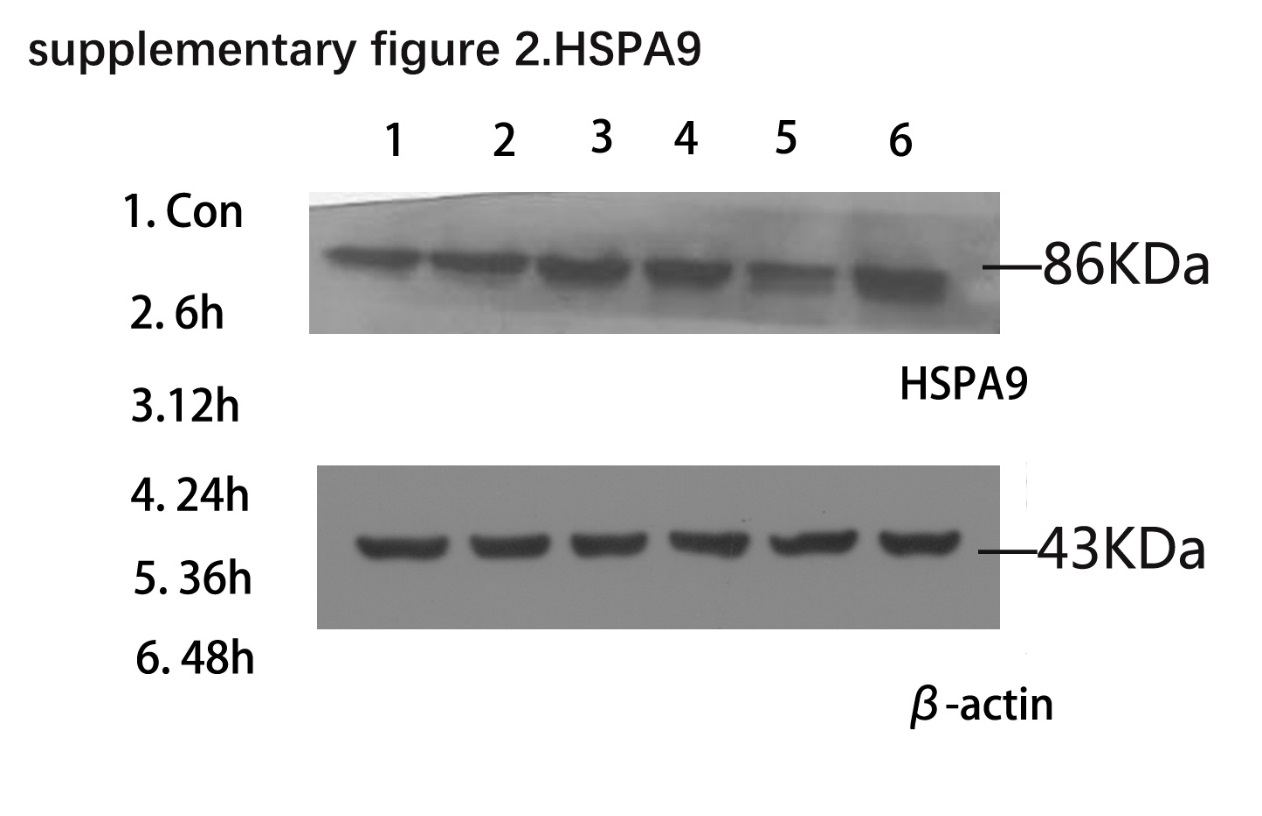


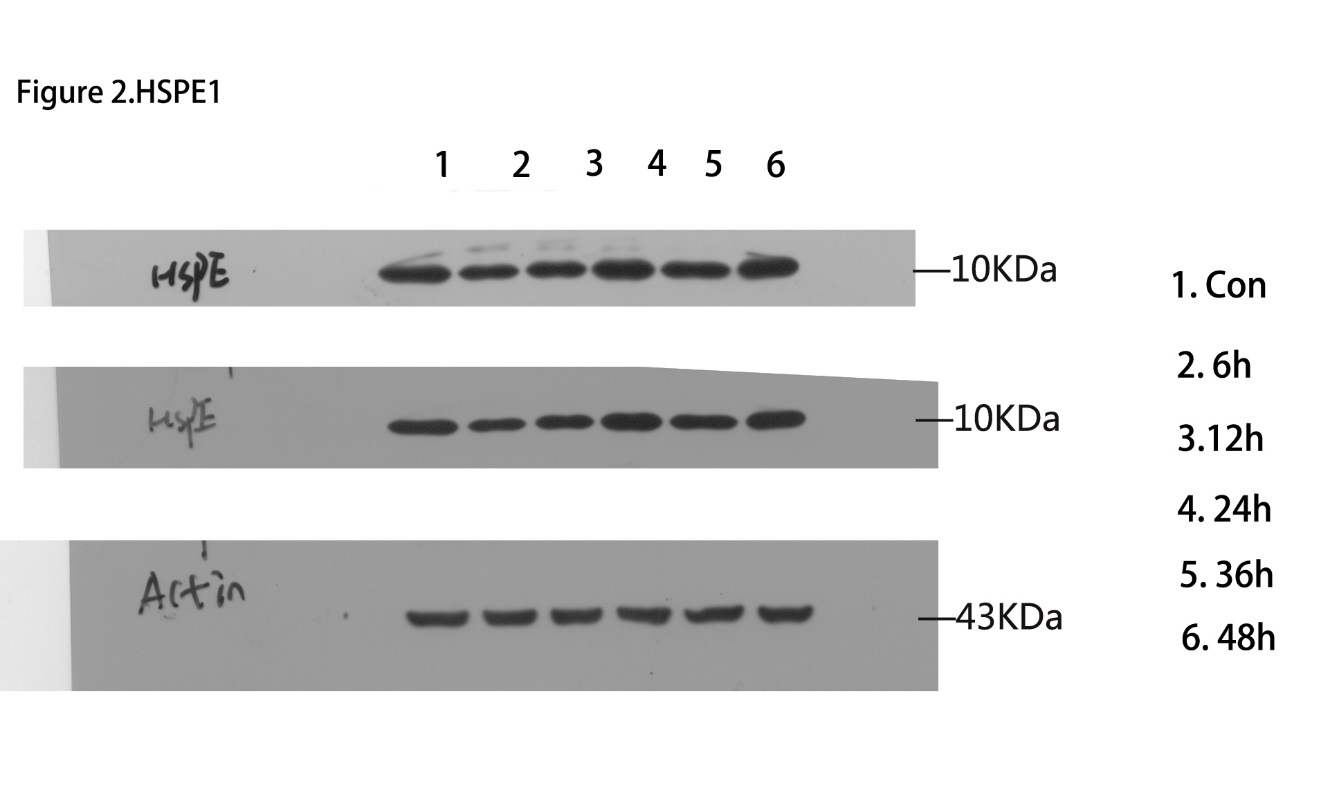


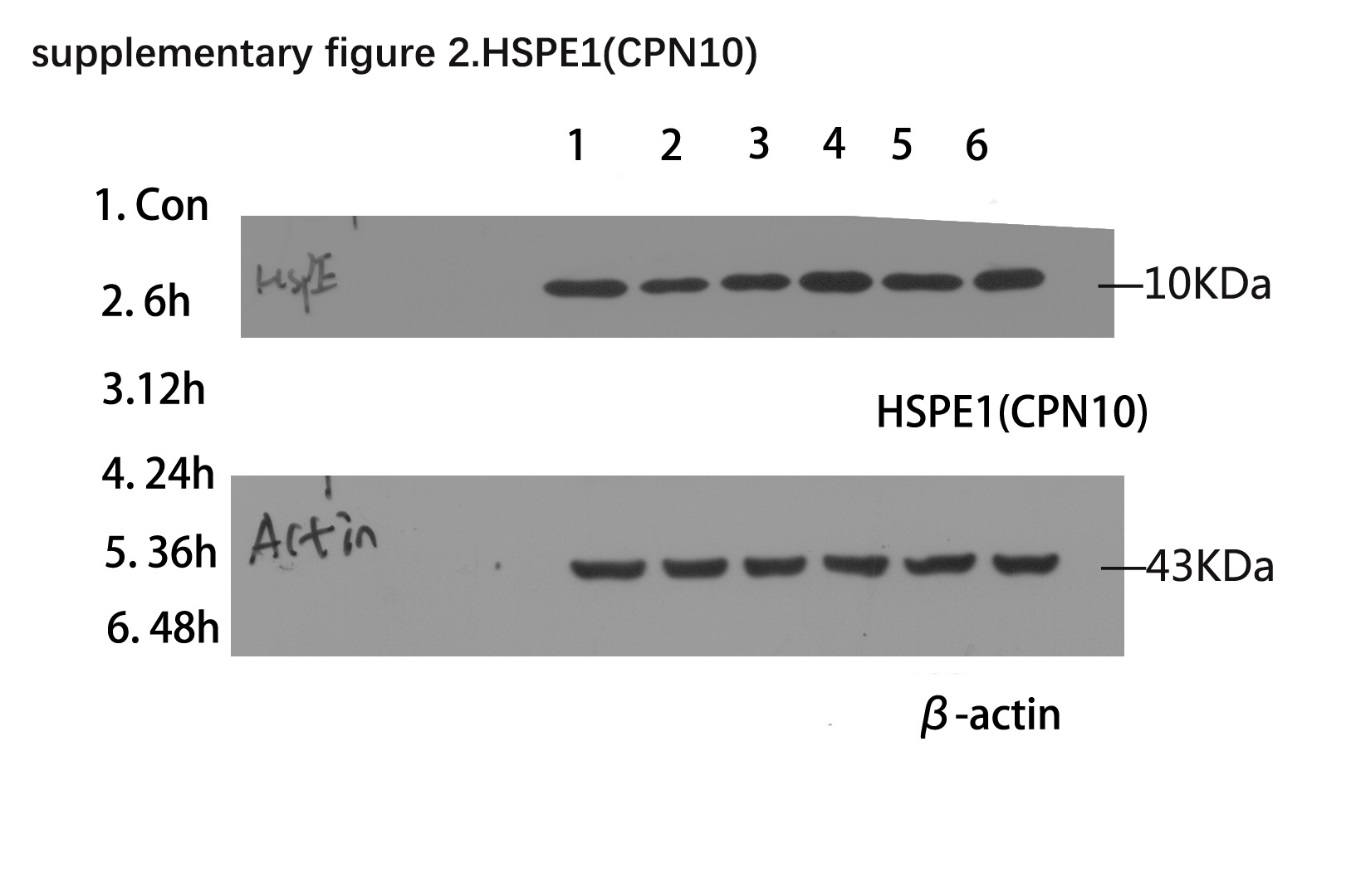


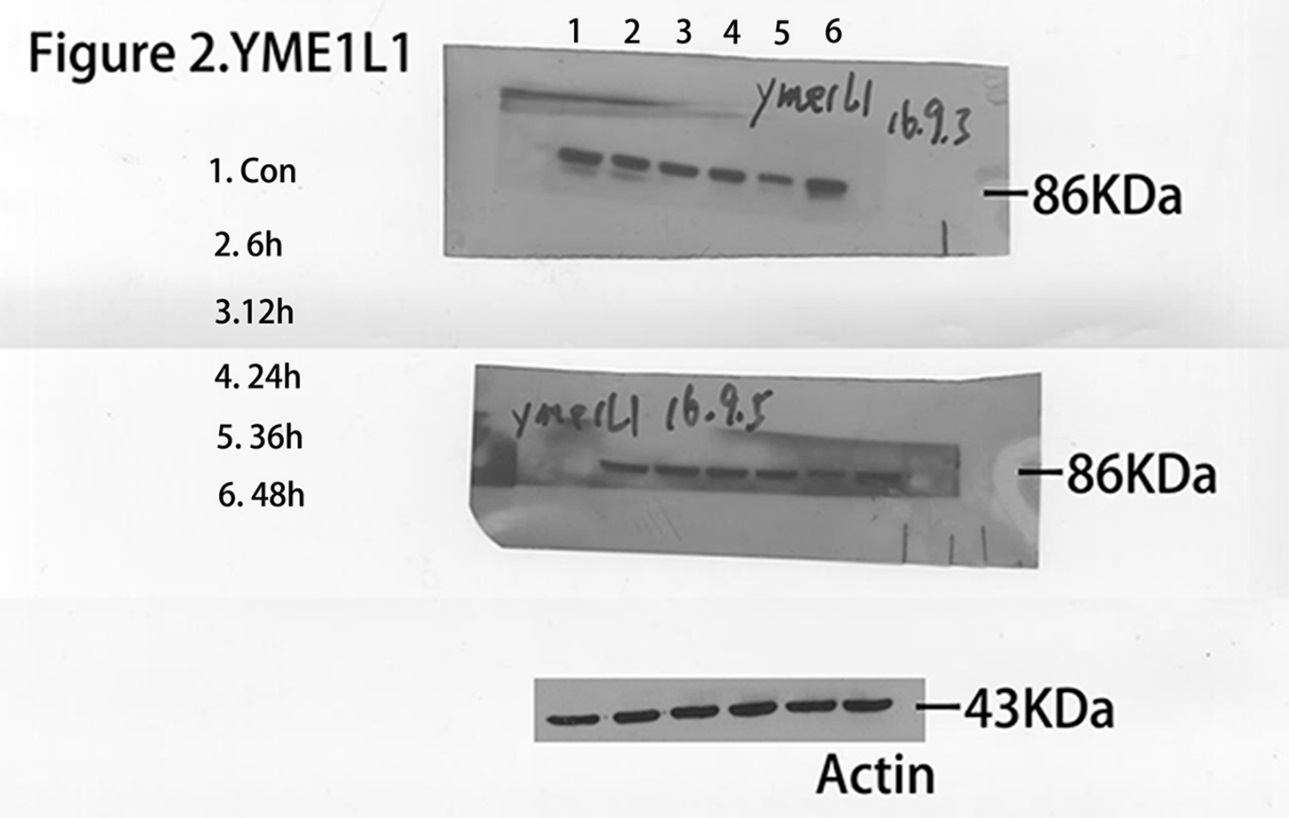


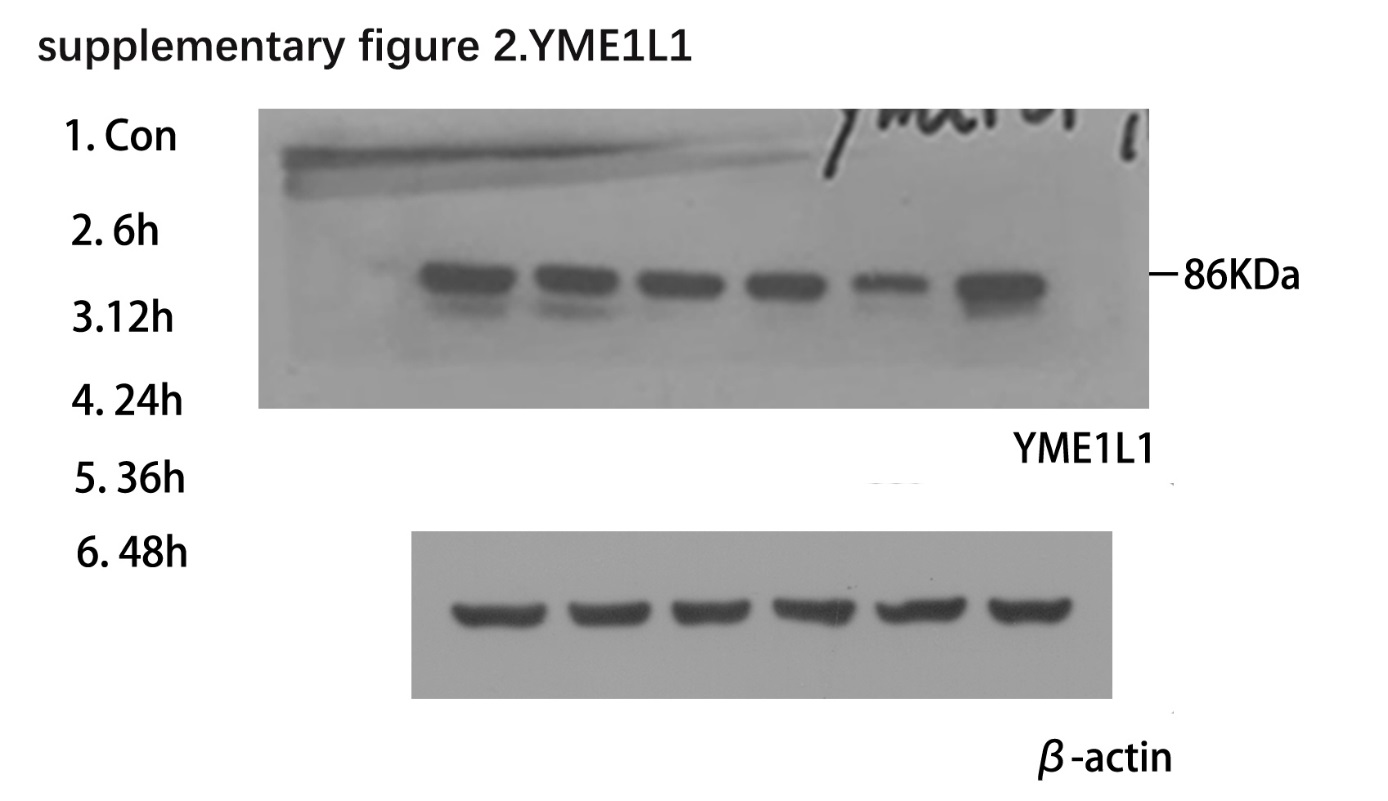


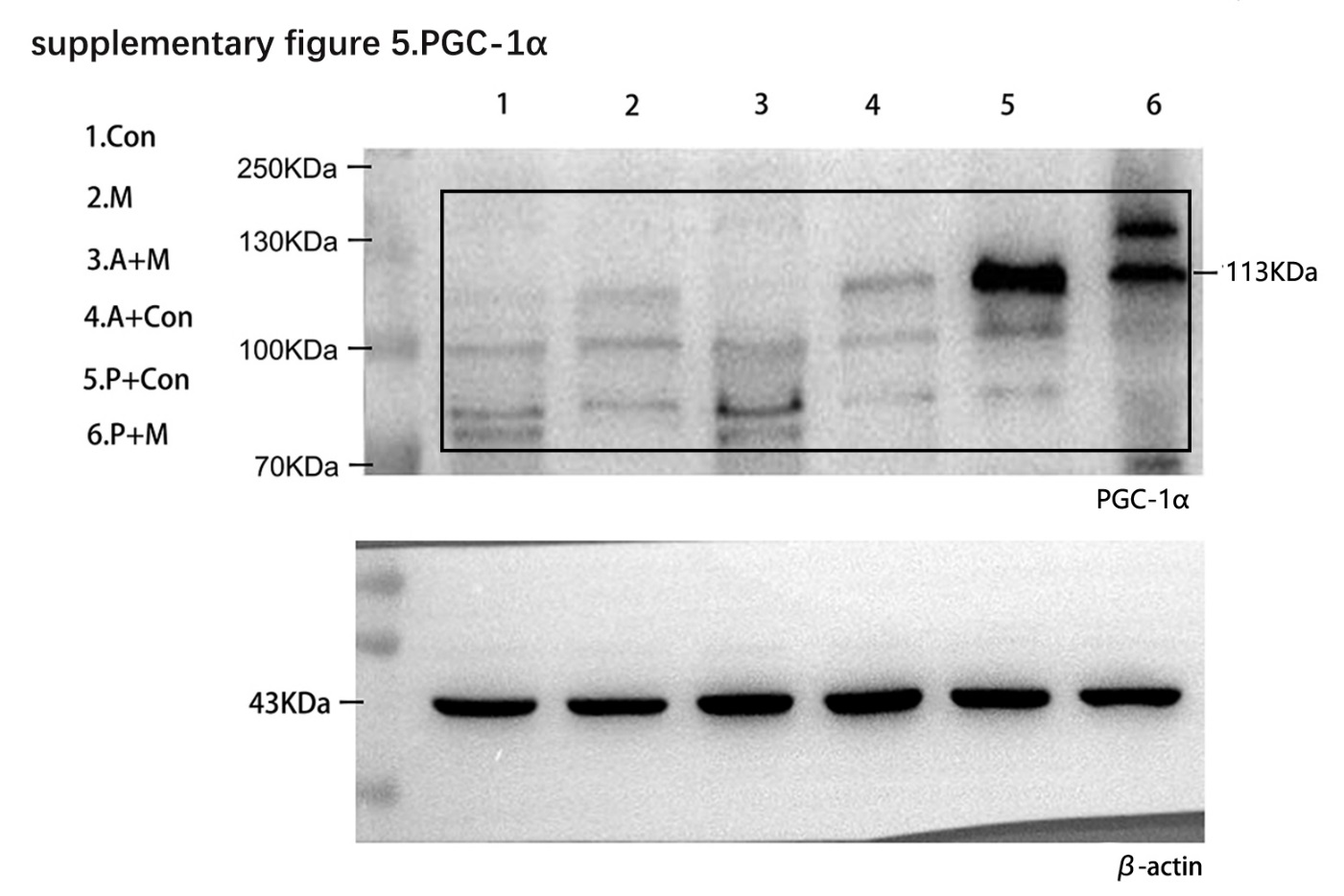


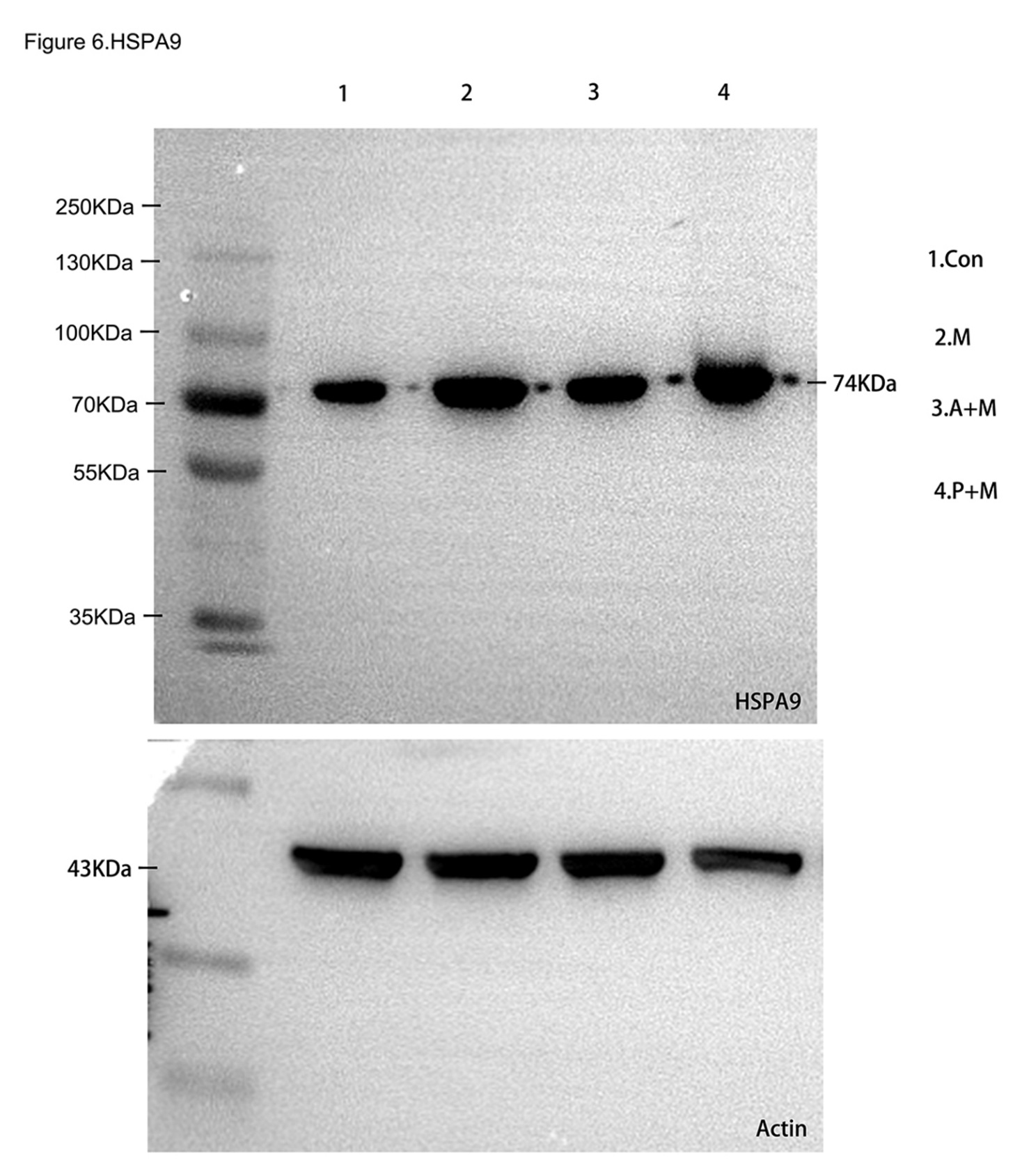


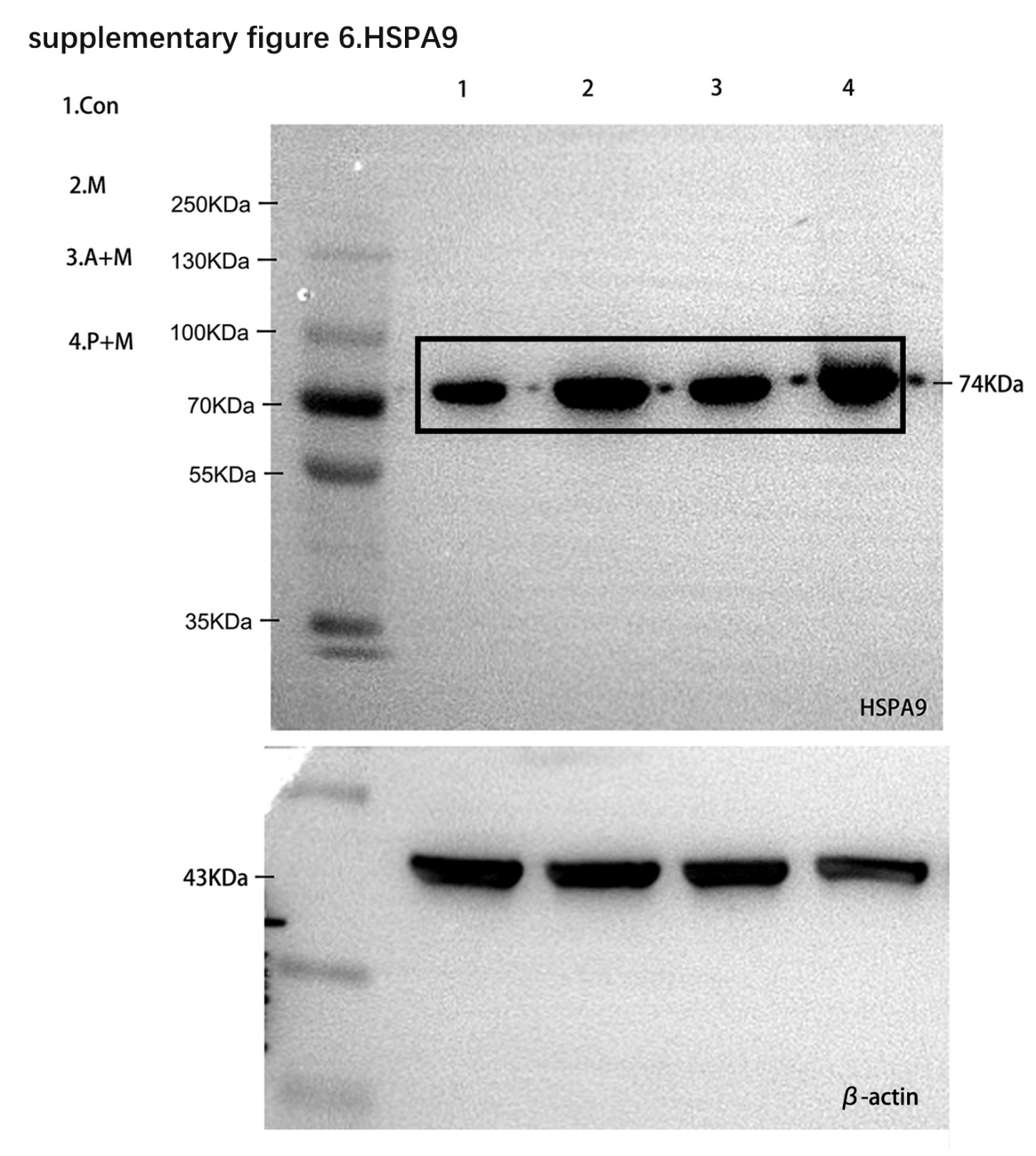


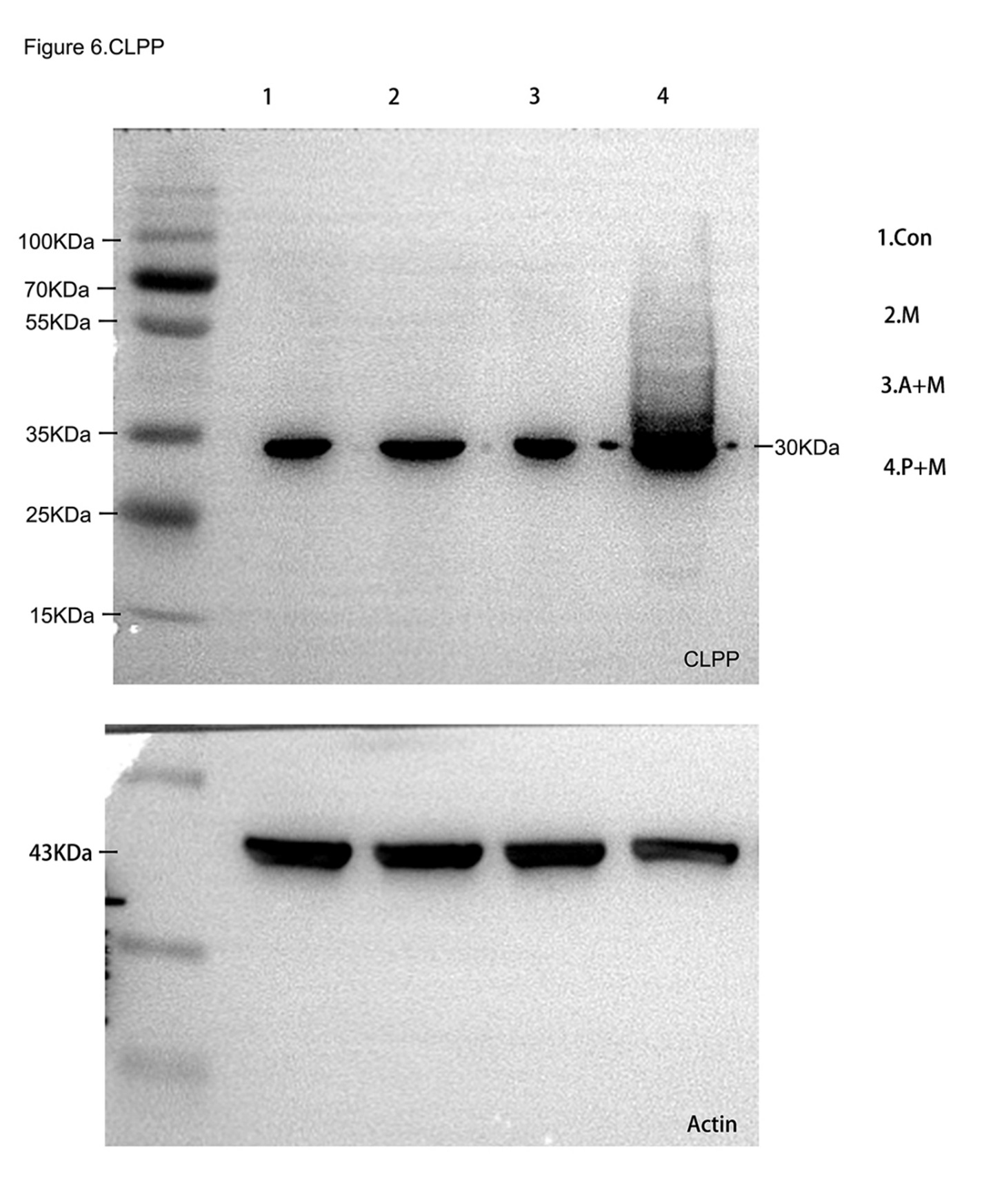


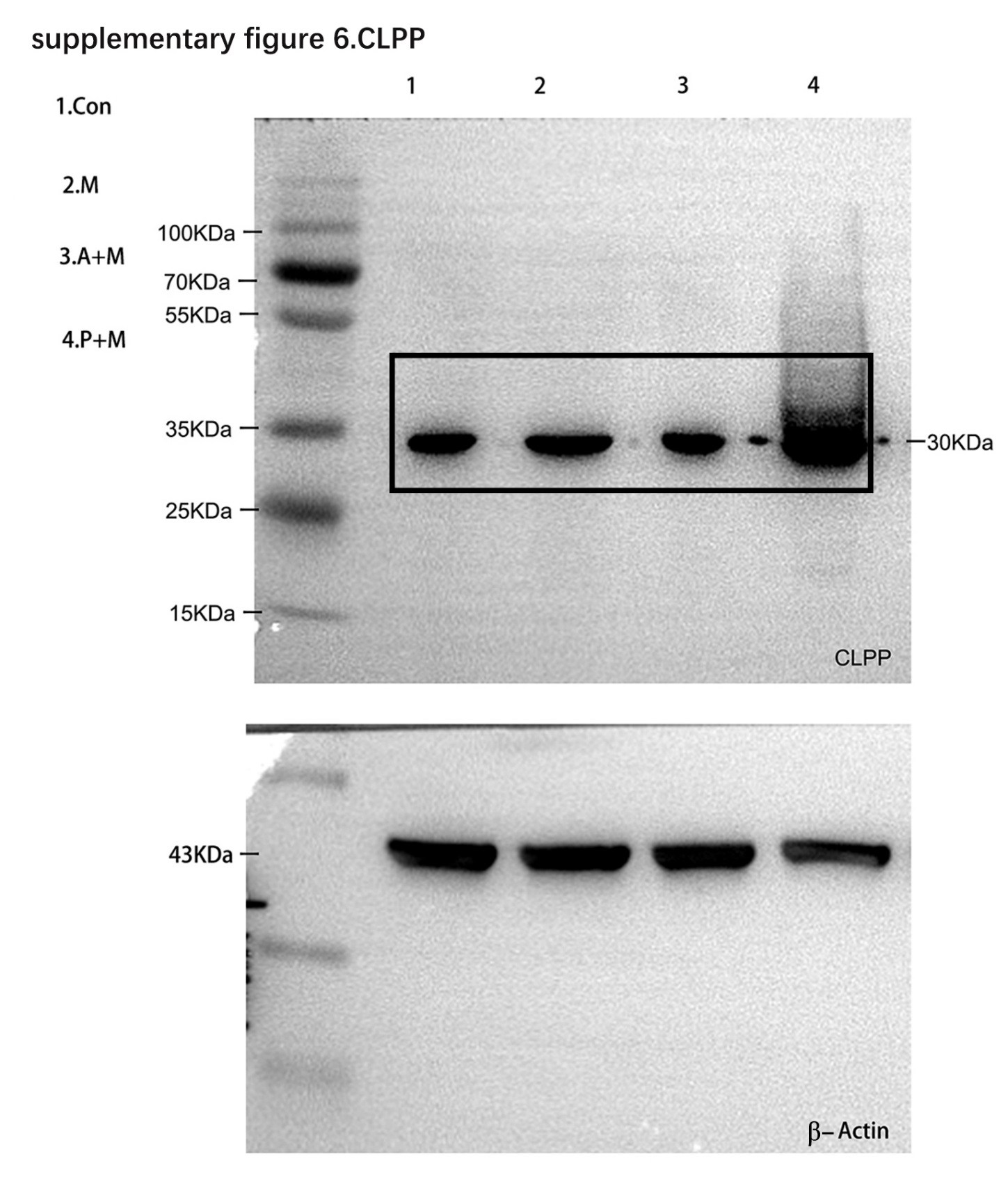


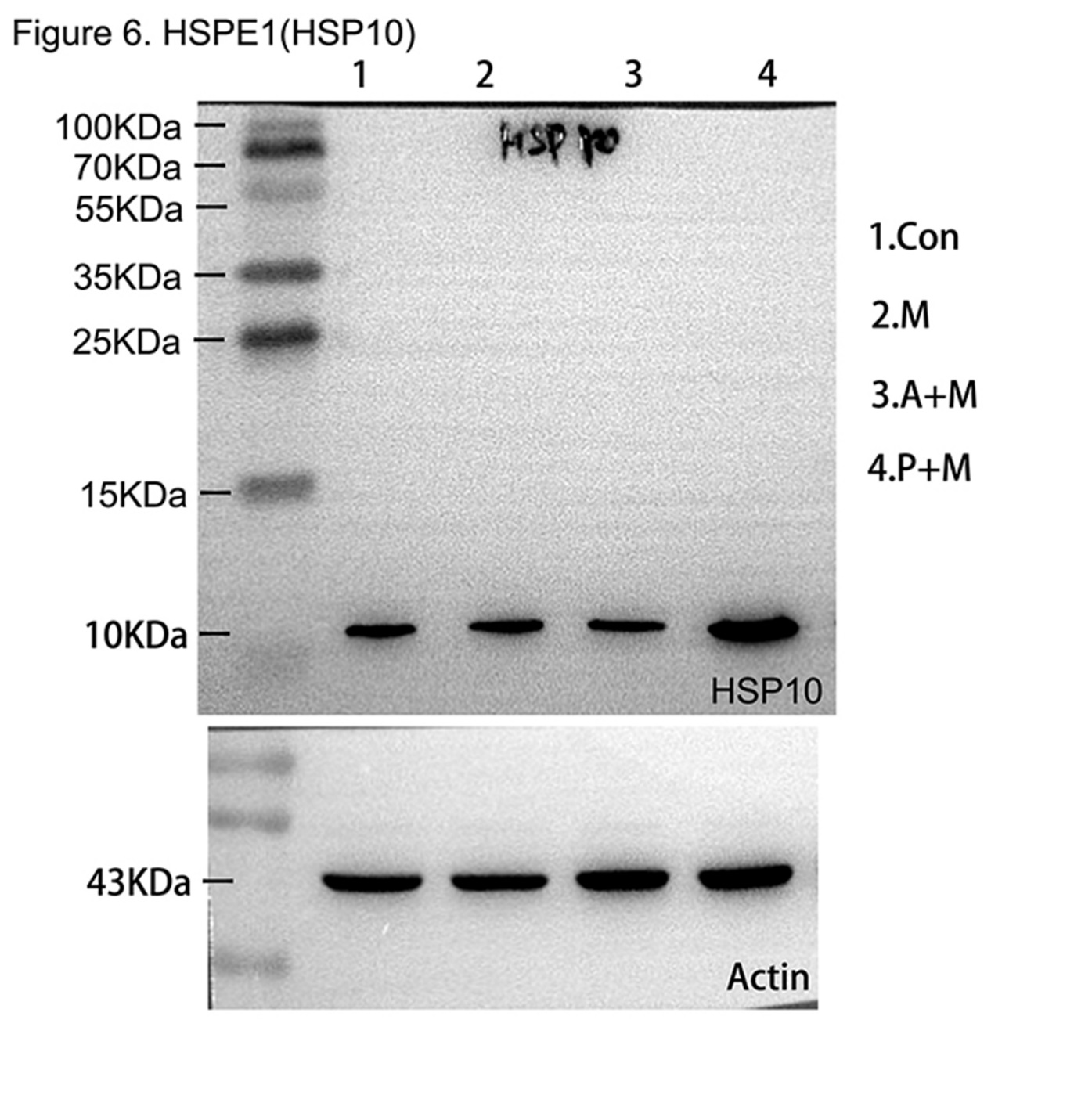


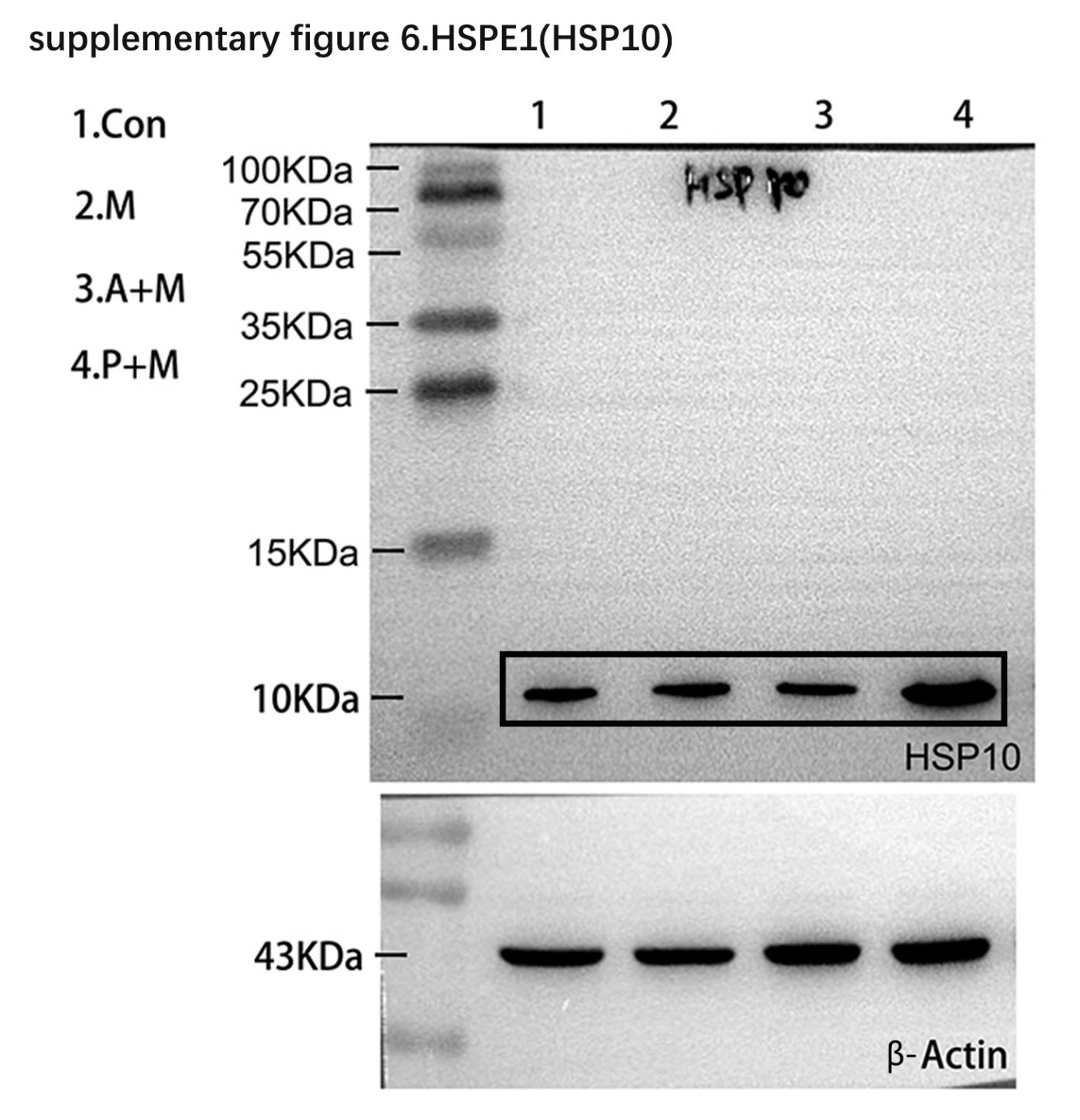


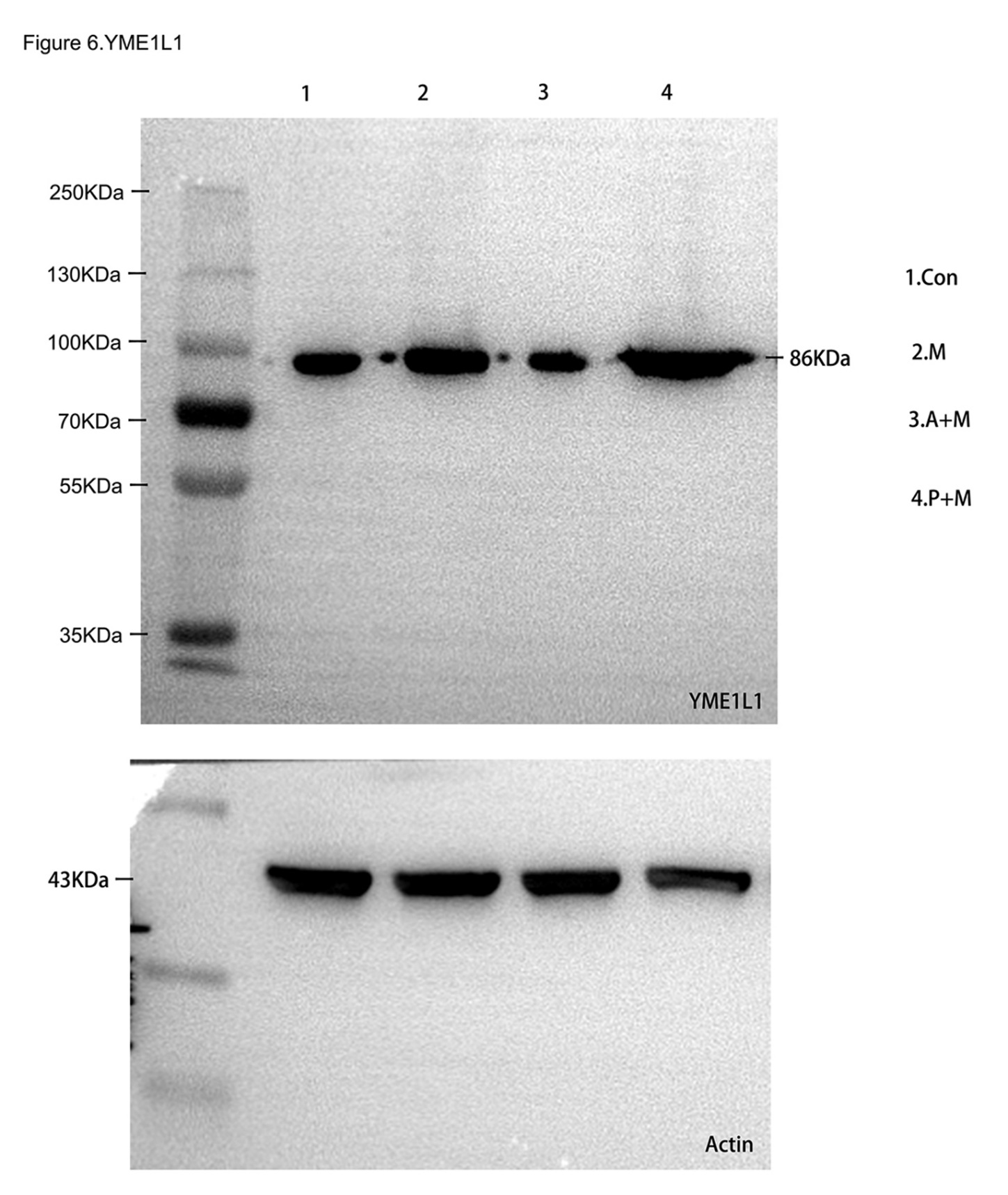


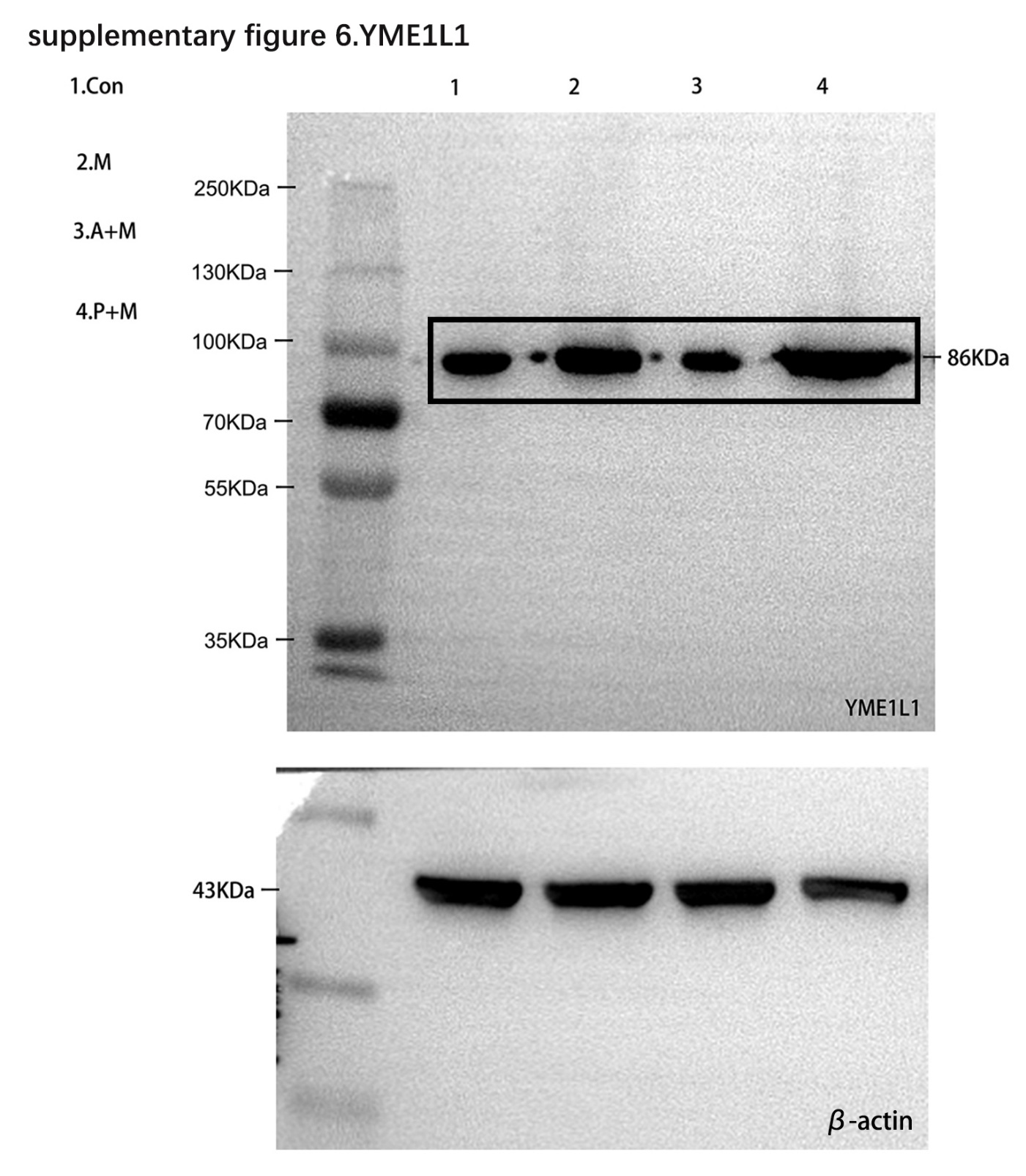


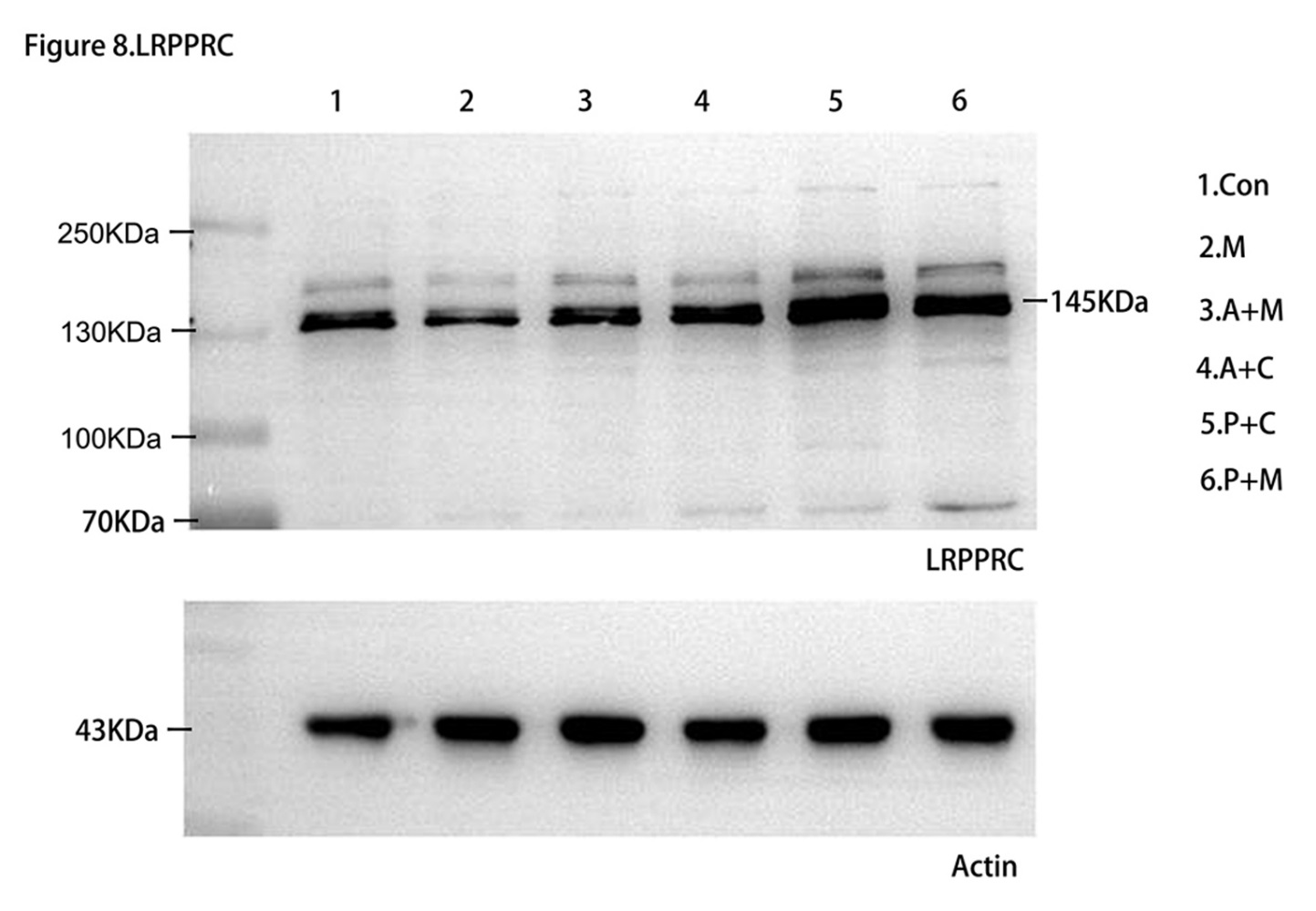


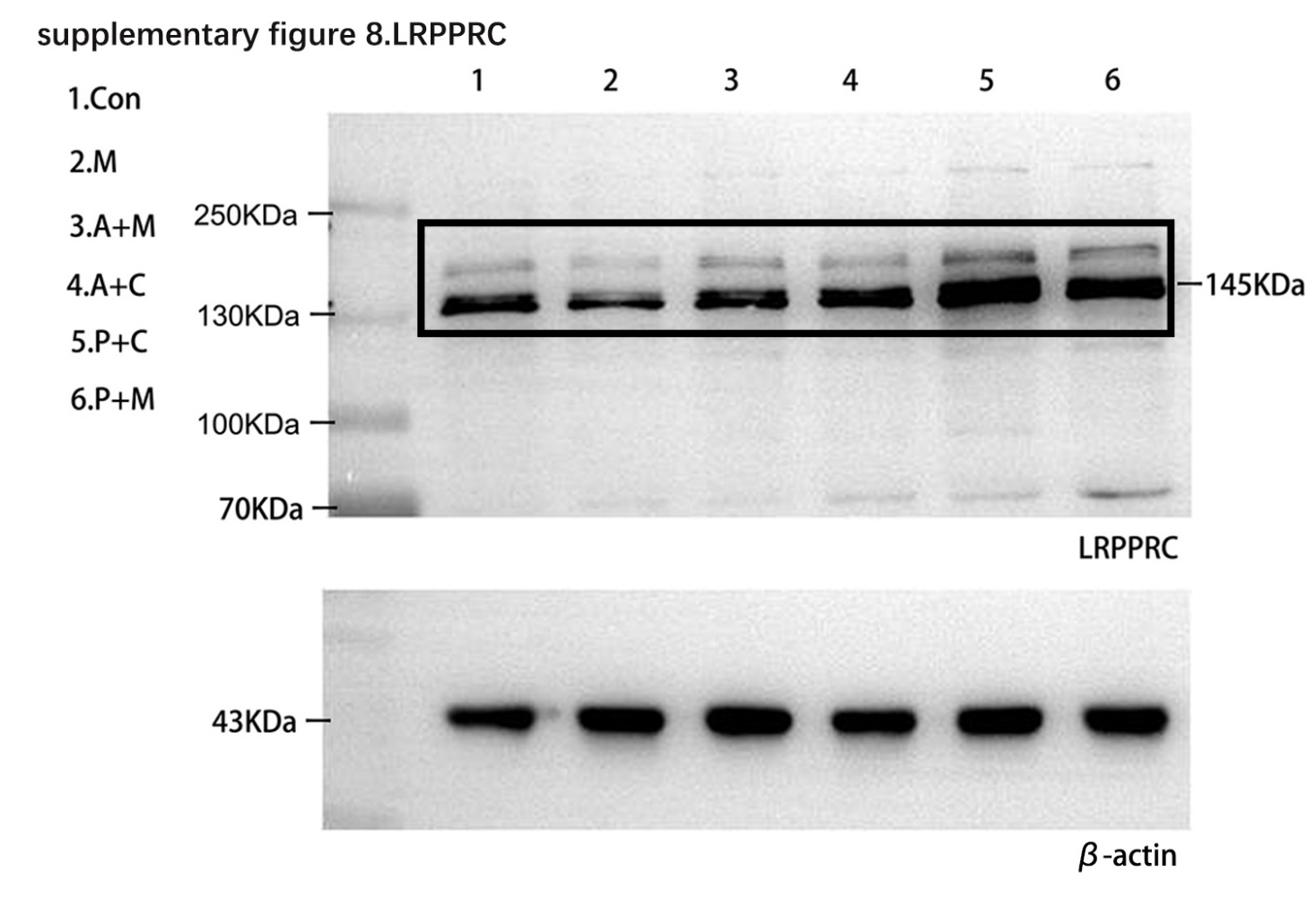

Supplement: Supplementary file 1 — Supplementary Information. [file 41598_2020_67229_MOESM1_ESM.docx]
